# *N*-Glycans and sulfated glycosaminoglycans contribute to the action of diverse Tc toxins on mammalian cells

**DOI:** 10.1371/journal.ppat.1009244

**Published:** 2021-02-04

**Authors:** Nan Song, Lihong Chen, Xingmei Ren, Nicholas R. Waterfield, Jian Yang, Guowei Yang

**Affiliations:** 1 Beijing Institute of Tropical Medicine, Beijing Friendship Hospital, Capital Medical University, Beijing, China; 2 Emergency and Critical Care Center, Beijing Friendship Hospital, Capital Medical University, Beijing, China; 3 NHC Key Laboratory of Systems Biology of Pathogens, Institute of Pathogen Biology, Chinese Academy of Medical Sciences and Peking Union Medical College, Beijing, China; 4 Warwick Medical School, Warwick University, Coventry, United Kingdom; Purdue University, UNITED STATES

## Abstract

Tc toxin is an exotoxin composed of three subunits named TcA, TcB and TcC. Structural analysis revealed that TcA can form homopentamer that mediates the cellular recognition and delivery processes, thus contributing to the host tropism of Tc toxin. *N*-glycans and heparan sulfates have been shown to act as receptors for several Tc toxins. Here, we performed two independent genome-wide CRISPR-Cas9 screens, and have validated glycans and sulfated glycosaminoglycans (sGAGs) as Tc toxin receptors also for previously uncharacterized Tc toxins. We found that TcdA1 form Photorhabdus luminescens W14 (TcdA1^W14^) can recognize *N*-glycans *via* the RBD-D domain, corroborating previous findings. Knockout of *N*-glycan processing enzymes specifically blocks the intoxication of TcdA1^W14^-assembled Tc toxin. On the other hand, our results showed that sGAG biosynthesis pathway is involved in the cell surface binding of TcdA2^TT01^ (TcdA2 from *P*. *luminescens* TT01). Competition assays and biolayer interferometry demonstrated that the sulfation group in sGAGs is required for the binding of TcdA2^TT01^. Finally, based on the conserved domains of representative TcA proteins, we have identified 1,189 putative TcAs from 1,039 bacterial genomes. These TcAs are categorized into five subfamilies. Each subfamily shows a good correlation with both genetic organization of the TcA protein(s) and taxonomic origin of the genomes, suggesting these subfamilies may utilize different mechanisms for cellular recognition. Taken together, our results support the previously described two different binding modalities of Tc toxins, leading to unique host targeting properties. We also present the bioinformatics data and receptor screening strategies for TcA proteins, provide new insights into understanding host specificity and biomedical applications of Tc toxins.

## Author summary

The Toxin complexes, also referred to as Tc toxins, are a family of A_5_BC exotoxins widely distributed among Gram-negative and positive bacteria. First identified in Entomopathogenic bacteria as key virulence factors to combat insect hosts, putative Tc toxin loci are also encoded by a range of human pathogens such as *Salmonella* and *Yersinia*. Previous studies indicated that several Tc toxins can target invertebrate and vertebrate cells *via* binding with *N*-glycans and heparan sulfates. Here our genome-wide CRISPR-Cas9 screens validated that different Tc toxins utilized distinct receptors for the adhesion to their targets, which is determined by TcA homopentamer. For example, TcdA1 from *Photorhabdus luminescens* W14 (TcdA1^W14^) relies on *N*-glycan binding to exert its toxic effects, while sulfate groups of sulfated glycosaminoglycans are critical for the cell targeting of other TcAs such as TcdA2^TT01^ (TcdA2 from *P*. *luminescens* TT01). Consistent with the previously described different binding modalities of Tc toxins, our results confirm that the receptor selectivity of TcAs contribute to the cellular tropism of Tc toxins. Furthermore we has also identified 1,189 TcA homologues and categorized them into five subfamilies. Each TcA subfamily shows a good correlation with the taxonomic origin of the genomes, suggesting these subfamilies are linked to diverse host tropisms *via* different binding modalities. Together, our findings provide mechanistic insights into understanding host specificity of distinct Tc toxins and the development of therapeutics for Tc toxin-related infections, as well as the adaptation of Tc-injectisomes as potential biotechnology tools and pest-control weapons.

## Introduction

Bacterial pathogens deploy a range of toxins to combat the host immune system, and favor the microbial infection [1]. These toxins can manipulate host cell signaling pathways, induce cell death by damaging the cytoplasmic membrane or cytoskeleton, or modify host proteins such as Rho GTPase [2–4]. Well-characterized toxins include the anthrax toxin from *Bacillus anthracis*, Exotoxin A and Exotoxin B produced by *Clostridium difficile*, *Staphylococcus aureus*-derived staphylococcal enterotoxin, *etc*. [5–7].

The insecticidal Tc toxins, first identified in entomopathogenic bacteria, are hetero-multimeric exotoxins composed of TcA, TcB and TcC subunits [8]. High-resolution structures of example Tc toxins reveal that the TcA assembles as a bell-shaped pentamer which mediates target cell recognition and effector domain delivery, while TcB and TcC form a cocoon-like structure that catalyzes the autocleavage of the toxic TcC C-terminal domain [9–14]. The released TcC C-terminal domain, is translocated into the target cell cytoplasm by an injectosome-like mechanism, leading to the toxic effect [9,15–17]. For example, PTC3 from *Photorhabdus luminescens* W14, composed of TcdA1, TcdB2 and TccC3, induces actin polymerization in *G*. *mellonella* hemocytes, due to the adenosine diphosphate (ADP)-ribosyltransferase activity of the TccC3 C-terminal fraction [15]. Moreover, only 0.5 nM of PTC3 is sufficient to cause the intoxication of target cells, indicating the Tc toxin functions as a potent virulence factor to facilitate bacterial infections [9].

Tc toxin homologues can encode a variety of diverse putatively toxic domains, thus can be classified as a polymorphic toxin system (PTS). From publicly available bacterial genomes in GenBank, our previous study identified 1,608 Tc loci encoded in 1,421 bacterial genomes, which are widely distributed among Gram-negative and positive bacteria (http://www.mgc.ac.cn/dbTC/). Further analysis indicated that the Tc toxin TcC subunit harbors a hypervariable region (HVR) at its C-terminus, representing the toxic “bullet” of the system. Nearly 200 distinct HVR clusters have been characterized among the 2,528 TcC protein homologues. Moreover, in addition to the previously mentioned entomopathogenic bacteria, Tc toxins are widely encoded in many diverse pathogenic bacteria, such as *Salmonella enterica* and *Yersinia pestis* [11,18,19]. This indicates that Tc toxins represent a polymorphic toxin system that can target various eukaryotic hosts and facilitate diverse pathogenic mechanisms.

Though the high-resolution cryo-EM structure and phylogenetic distribution of Tc toxins have been studies, little is known about the cellular recognition specificities of Tc toxins. As host cell-surface glycans have been previously reported to serve as ligands for various toxins, two independent studies utilized comprehensive glycan microarray screens in attempts to identify potential receptor ligands that may contribute to the cell surface binding Tc toxins [20,21]. Piper *et al*. showed that YenTc, as well as the Chi1 and Chi2 subunits, can bind with a range of glycan structures that incorporate galactose, glucose, *N*-acetylgalactosamine and *N*-aceytylglucosamine motifs, suggesting that glycans play an important role in cellular recognition by YenTc [20]. Meanwhile, Roderer *et al*. focused on the screening of receptors of different TcAs from insect and human pathogens, demonstrating that *Photorhabdus luminescens* W14-derived TcdA1 can bind with glycan epitopes such as trisaccharide Lewis X, the tetrasaccharides Lewis Y and sialyl-Lewis X. They also reported that both *Morganella morganii* TcdA4 and *Xenorhabdus nematophila* XptA1 interact with negatively charged heparins [21]. Although both these studies characterized the glycans that interact with Tc toxins, it is still unclear which host factors/pathways are critical to allow intoxication by Tc toxins. To elucidate this issue, we performed the genome-wide CRISPR-Cas9 screens using Tc toxin PTC3-induced cytotoxicity, and determined two sets of host genes that are involved in the action of different Tc toxins. In addition, based on the Tc database we previously established, we have analyzed all available TcA homologues from both entomopathogenic and human-pathogenic bacteria, and classified them into 5 subfamilies, which show a good correlation with the taxonomic origin of the genomes. Taken together, our study provides an effective strategy for future screening of Tc toxin receptors, and that analysis of the TcA database offers the potential to decipher the host specificity and possible future biomedical applications of Tc toxins.

## Results

### *N*-linked Glycans are required for the cellular recognition by the TcdA1^W14^ pentamer

Though first identified as an insecticidal toxin, the activities of Tc toxins have been studied in a variety of mammalian cells [9,15,18]. For example, PTC3^W14^, a Tc toxin derived from entomopathogenic bacteria *P*. *luminescens* W14, can target both HeLa and HEK293T cells, and induce aberrant actin polymerization, suggesting that the receptor(s) of this Tc toxin are ubiquitously expressed in both invertebrate and vertebrate cells [9,15]. PTC3^W14^ is composed of TcdA1 (hereinafter referred to as TcdA1^W14^), TcdB2 and TccC3, corresponding to the TcA, TcB and TcC subunits respectively [15]. Considering the TcdA1^W14^ pentamer has been proposed as mediating toxin binding to cell surfaces, we carried out a genome-wide CRISPR–Cas9 mediated knock-down screening with the aim of identifying the host factor(s) involved in TcdA1^W14^ interaction.

**Fig 1:**
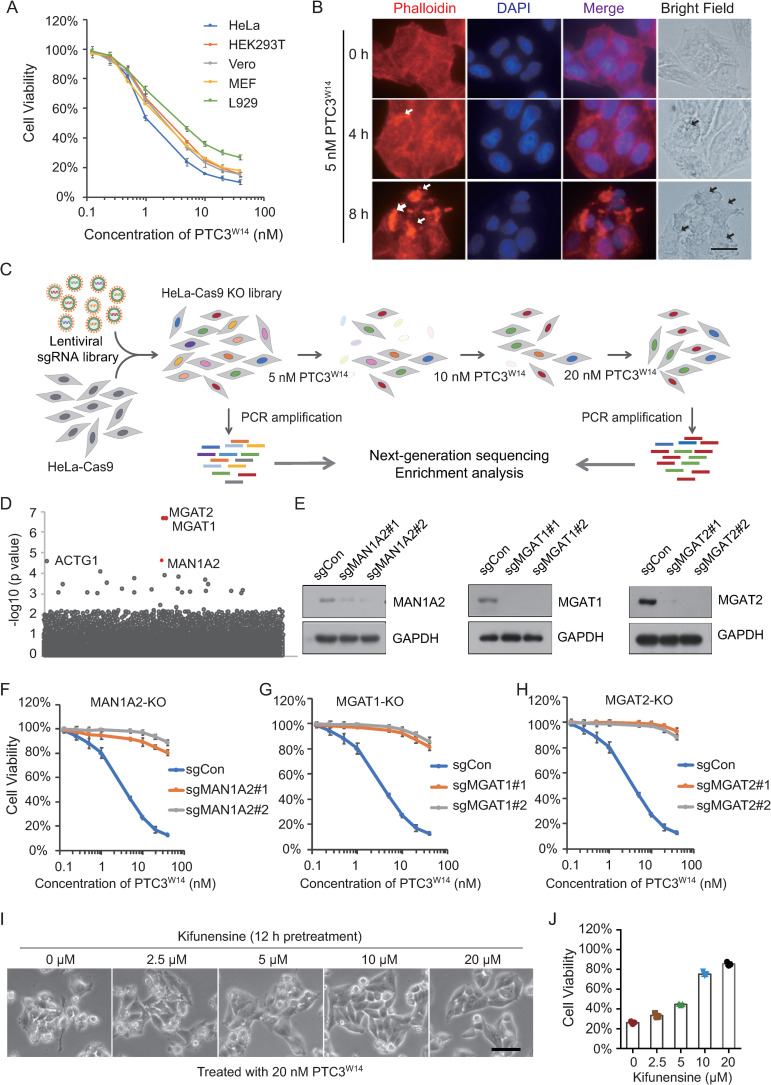
*N*-linked Glycans are required for the cell targeting of the TcdA1^W14^ pentamer. (A) Five mammalian cell lines were treated with the indicated doses of PTC3^W14^ for 24 h. Cell viability was measured using CCK-8 assays. The PTC3^W14^ concentration that leads to the death of 50% of cells is defined as IC_50_. (B) HeLa-Cas9 cells transduced with a non-targeting sgRNA were treated with 5 nM PTC3^W14^ for 0, 4 and 8 h. Cells were fixed, permeabilized and stained with Alexa 568-phalloidin (red) and DAPI (blue). Representative fluorescence and bright field micrographs are shown. White arrows indicate polymerized F-actin. Black arrows indicate membrane blebs. Scale bars, 5 μm. (C) Schematic drawing of the screening approach for host factors that are critical for the intoxication of PTC3^W14^. HeLa cells stably expressing Cas9 (HeLa-Cas9) were transduced with lentiviral GeCKO v.2 sgRNA libraries. These cells were exposed to increased doses of PTC3^W14^ (5, 10 and 20 nM). Cells that were not treated with PTC3^W14^ were served as controls. Total genomic DNA from 2×10^7^ selected and control cells was used for sequencing. The enriched sgRNAs were sequenced by NGS, followed by MAGeCK analysis. (D) Genes identified in the screens with PTC3^W14^ treatment were ranked based on to the MAGeCK *p* values. MAN1A2, MGAT1, and MGAT2 were identified as the key genes for PTC3^W14^ recognition of HeLa cells. (E) The efficiency of gene knockout was determined by Western blot analysis with the indicated antibodies. GAPDH served as the loading control. (F-H) MAN1A2-KO, MGAT1-KO, and MGAT2-KO HeLa-Cas9 cells were treated with the indicated doses of PTC3^W14^ for 24 h. Cell viability was measured using CCK-8 assays. (I-J) HeLa-Cas9-sgCon cells were pretreated with the indicated doses of Kifunensine for 12 h, and then exposed to 20 nM PTC3^W14^. Representative bright field micrographs were shown (I). Cell viability was measured using CCK-8 assays (J). All error bars indicate mean ± SD. Each of the experiments was repeated three times.

PTC3^W14^, assembled from TcdA1^W14^ and *P*. *luminescens* W14-derived functionally active TcdB2-TccC3 fusion proteins, was recombinantly expressed in *E*. *coli* as described previously (S1A Fig) [15]. Both gel filtration chromatography and negative-stain electron microscopy showed that PTC3^W14^ was successfully prepared (S1A–S1C Fig). We first assessed the sensitivity of a series of mammalian cell lines to PTC3^W14^ treatment. Five mammalian cells including HEK293T, HeLa, Vero, L929 and MEF, were exposed to a titration of PTC3^W14^ for 24 h, and the percentage of surviving cells was assayed by CCK-8. As shown in Fig 1A, all the tested cell lines are sensitive to PTC3^W14^. HeLa cells were one of the most sensitive lines, with an IC_50_ of approximately 1 nM. Therefore, we elected to use HeLa cells to perform the CRISPR/Cas9 knock-out screens. HeLa cells stably expressing Cas9 (HeLa-Cas9) were generated and transduced with a lentiviral single guide RNA (sgRNA) library (GeCKO v.2), which targets 19,050 protein-coding genes and 1,864 microRNAs in the human genome [22]. Both HeLa-Cas9 cells and HeLa-Cas9 cells transduced with a non-targeting sgRNA (HeLa-Cas9-sgCon) showed similar sensitive to PTC3^W14^ when compared with wild-type HeLa cells (S1D Fig). We also noted that treatment with 5 nM PTC3^W14^ for 8 h is sufficient to induce robust actin polymerization and membrane blebbing in HeLa-Cas9-sgCon cells (Fig 1B). Following three rounds of selection with PTC3^W14^ (at 5, 10 and 20 nM, Fig 1C), the surviving transduced HeLa-Cas9 cells exhibited robust resistance to PTC3^W14^ intoxication, suggesting that key genes involved in the PTC3^W14^ mode of action had been successfully ablated.

The relevant sgRNA targeted genes were identified using next-generation sequencing (NGS) of the population of PTC3^W14^-resistant cells, and ranked according to the *p* value of MAGeCK analysis (y axis) (Fig 1D and S1 and S2 Tables). The majority of top-ranked genes fall into the *N*-linked Glycan (*N*-glycan) synthesis pathway, and include enzymes that convert oligo-mannose to galactose-containing complex *N*-linked glycans, such as Mannosyl-oligosaccharide 1,2-α-mannosidase 1B (MAN1A2), α-1,3-mannosyl-glycoprotein 2-β-N-acetylglucosaminyltransferase (MGAT1) and α-1,6-mannosyl-glycoprotein 2-β-N-acetylglucosaminyltransferase (MGAT2) [23]. To validate the results of the PTC3 screening, we next generated MGAT1-, MGAT2- and MAN1A2-KO HeLa cells, respectively (Fig 1E). All these KO cell lines showed increased resistance to PTC3^W14^ treatment compared with control cells (Figs 1F–1H and S1D). Furthermore, we also utilized kifunensine, as a potent and selective inhibitor of class I α-mannosidases, to pharmacologically block the synthesis of *N*-glycans without interfering with cell viability [24,25]. Consistently, pretreatment with kifunensine was also shown to significantly inhibit intoxication by PTC3^W14^ (Fig 1I and 1J). In addition, knockout of *O*-linked glycan (*O*-Glycan) genes such as core 1 glycoprotein-*N*-acetylgalactosamine 3-β-galactosyltransferase 1 (C1GalT1) did not show any effect on PTC3^W14^-induced membrane blebbing and cell death (S1E and S1F Fig). Taken together with the structural data showing that glycan interacts with TcdA1^W14^ [21], these data demonstrated that *N*-glycans are indeed required for the cell targeting of TcdA1^W14^ pentamer, mediating the cellular recognition process.

### The receptor binding domain-D (RBD-D) of TcdA1^W14^ mediates the interaction with *N*-glycans

**Fig 2:**
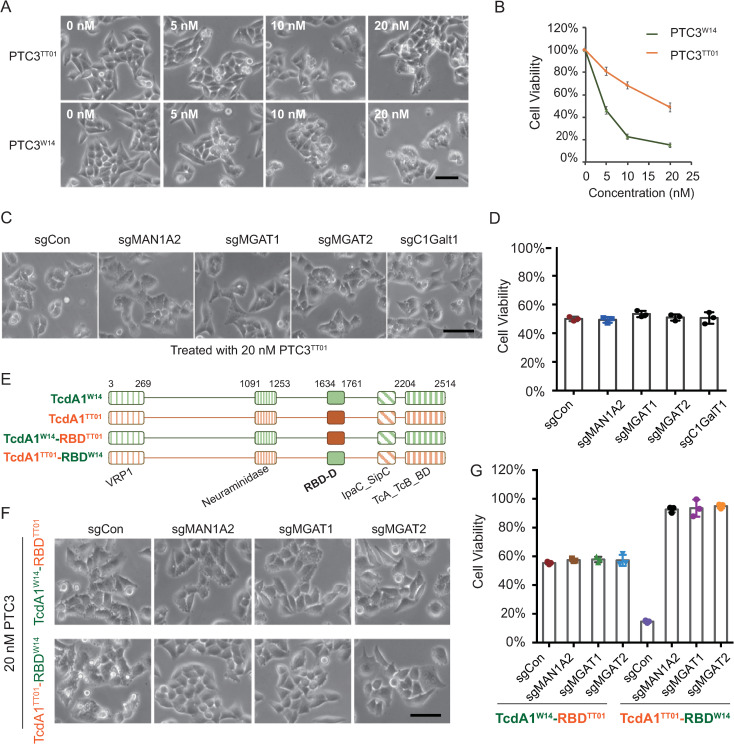
The TcA receptor binding domain of TcdA1^W14^ is required for *N*-glycan-mediated intoxication. (A-B) Representative bright field micrographs of indicated HeLa-Cas9 cells treated with indicated doses of PTC3^TT01^ or PTC3^W14^ (A). Cell viability was measured using CCK-8 assays and shown in (B). (C-D) MAN1A2-KO, MGAT1-KO, MGAT2-KO, C1GalT1-KO, and HeLa-Cas9-sgCon cells were exposed to 20 nM PTC3^TT01^. Representative bright field micrographs (C) and the effects on cell viability (D) were shown. (E) Schematic drawing of indicated TcAs. The N-terminal VRP1 domain (PF03538), the middle Neuraminidase domain (PF18413) and the C-terminal TcA_TcB_BD domains (PF18276) were shown. The RBD-D domains derived from TcdA1^TT01^ and TcdA1^W14^ are depicted in orange and green, respectively. (F-G) MAN1A2-KO, MGAT1-KO, MGAT2-KO, and HeLa-Cas9-sgCon cells were exposed to 20 nM of the indicated toxins. Representative bright field micrographs were shown (F). Cell viability was measured using CCK-8 assays (G). All error bars indicate mean ± SD. Each of the experiments was repeated three times.

We next investigated whether *N*-glycans mediate the cell binding of other TcA homologues. Based on the sequence alignment, we found that TcdA1 from *P*. *luminescens* TT01 (hereinafter referred to as TcdA1^TT01^), shares significant similarity with TcdA1^W14^, with the exception of the receptor binding domain-D (RBD-D), a putative TcA receptor binding domain (also referred to as TcA_RBD, Pfam:PF18518, 1637–1772 aa of TcdA1^TT01^) (S2A Fig). We therefore purified the Tc toxin, PTC3^TT01^, which is assembled using the TcdA1^TT01^ and *P*. *luminescens* W14-derived TcdB2-TccC3 fusion proteins (S2B Fig). Both actin polymerization and membrane blebbing was again observed in PTC3^TT01^-treated HeLa-Cas9 cells, although it was less pronounced when compared with TcdA1^W14^-assembled PTC3^W14^ (Figs 2A, 2B and S2C). Intriguingly, neither knockout of glycan-related genes nor kifunensine treatment was able to block intoxication by PTC3^TT01^ (Figs 2C and 2D and S2D and S2E), suggesting that the cellular recognition of PTC3^TT01^ is independent of *N*-glycans. Considering that RBD-D is the major structural difference between TcdA1^W14^ and TcdA1^TT01^, these data indicated that the RBD-D of TcdA1^W14^ is directly responsible for *N*-glycans-dependent intoxication, consistent with the previously report showing Lewis antigens can bind to RBD-D [21].

To examine whether RBD-D is sufficient for *N*-glycans-dependent intoxication, we generated a mutated TcdA1^TT01^ by replacing the RBD-D with that of TcdA1^W14^, and *vice versa* (Figs 2E and S3A–S3D). This mutated TcdA1^TT01^ (TcdA1^TT01^-RBD^W14^) was able to trigger robust membrane blebbing in control HeLa cells comparable to that seen for the wild-type TcdA1^W14^ complex (Figs 2F and S3E). Importantly, it was also not able to intoxicate *N*-glycan-deficient cells (Figs 2G and S3F). In contrast, the mutated TcdA1^W14^ (TcdA1^W14^-RBD^TT01^), which encodes the RBD-D of TcdA1^TT01^, showed equal toxigenic effects in both control HeLa cells (sgCon) and *N*-glycan-deficient cells (Figs 2G and S3F). Taken together, these results confirm that the RBD-D of TcdA1^W14^ can specifically mediate the *N*-glycans-dependent intoxication.

### The Trp1721 and Lys1738 of TcdA1^W14^ RBD-D are critical for *N*-glycan-dependent intoxication

**Fig 3:**
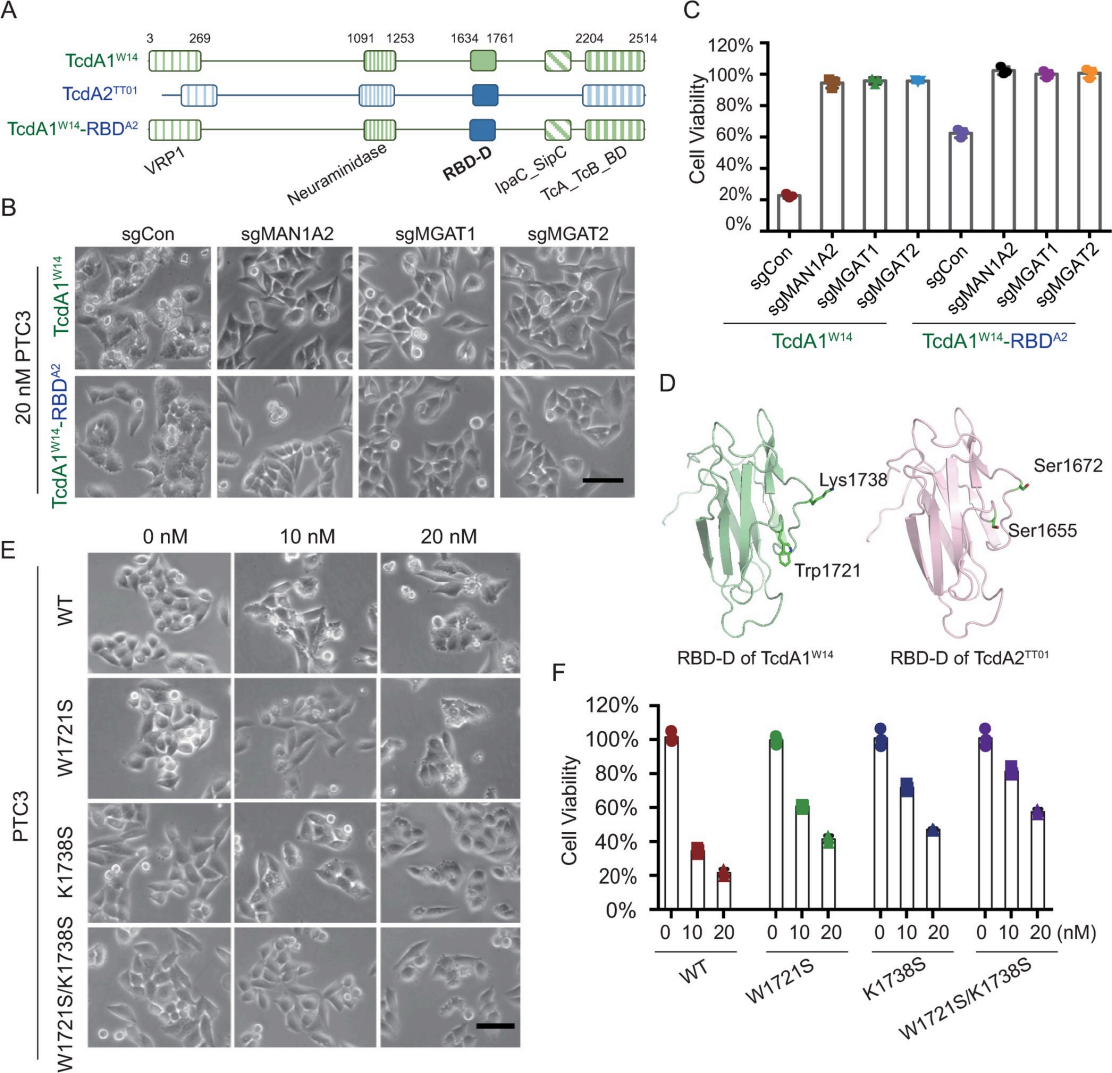
The Trp1721 and Lys1738 of TcdA1^W14^ are critical for *N*-glycan-mediated intoxication. (A) Schematic drawing of TcdA1^W14^, TcdA2^TT01^ and TcdA1^W14^-RBD^A2^. The RBD-D domains derived from TcdA1^W14^ and TcdA2^TT01^ are depicted in green and blue, respectively. (B-C) MAN1A2-KO, MGAT1-KO, MGAT2-KO, and HeLa-Cas9-sgCon cells were exposed to 20 nM of the indicated Tc toxins. Representative bright field micrographs (B) and the effects on cell viability (C) are shown. (D) Homology models of RBD-D domains. Swiss model program (ExPASy web server) was used to construct a homology model of the RBD-D of TcdA2^TT01^ by alignment with that of TcdA1^W14^ (PDB: 4O9Y). The Trp1721 and Lys1738 residues of TcdA1^W14^ (corresponding to Ser1655 and Ser1672 of TcdA2^TT01^) are indicated. (E-F) Representative bright field micrographs of indicated HeLa-Cas9 cells treated with the indicated doses of wild-type PTC3^W14^ and indicated mutants (W1721S, K1738S, W1721S/K1738S) (E). Cell viability was measured using CCK-8 assays and shown in (F). All error bars indicate mean ± SD. Each of the experiments was repeated three times.

Since the RBD-D of TcdA1^W14^ directly mediates binding to *N*-glycans [21], we screened other Tc toxin homologue sequences for those with the potential to bind *N*-glycans using TcA sequence alignment. We found that the RBD-D of TcdA2 from *P*. *luminescens* TT01 (TcdA2^TT01^) shares 88% identity with that of TcdA1^W14^ (S4A Fig). We therefore examined whether the RBD-D of TcdA2^TT01^ can also mediate *N*-glycan-dependent intoxication. To compare with the RBD-D of TcdA1^W14^, we generated a mutated TcdA1^W14^ by replacing the wild-type RBD-D with that of TcdA2^TT01^, referred to as TcdA1^W14^-RBD^A2^ (Fig 3A). As shown in Figs 3B and 3C and S4B and S4C, TcdA1^W14^-RBD^A2^ could also trigger membrane blebbing in control HeLa cells, but again not in *N*-glycan-deficient cells. However, the intoxication of TcdA1^W14^-RBD^A2^ was much weaker than that of TcdA1^W14^. These data suggest that the RBD-D of TcdA2^TT01^ does indeed possess the capacity for *N*-glycan binding, but that the toxic effect was not as strong as that of TcdA1^W14^, when in the sequence context of the rest of the TcdA1^W14^ protein. Alternatively, it is possible that the RBD-D of TcdA2^TT01^ sequence does not possess certain key amino acid residue(s) for mediating a strong interaction with *N*-glycans.

We next analyzed the RBD-D sequences of TcdA1^W14^ and TcdA2^TT01^, and found that most of the substitution differences did not lead to a change in the conserved amino acid properties, with the exception of two, Trp1721 and Lys1738 (Figs 3D and S4A). Both Trp1721 and Lys1738 of TcdA1^W14^ were substituted by Serine in TcdA2^TT01^. According to previous reports investigating the interaction between *N*-glycans and C-type lectins, the galactose residues of *N*-glycans can pack alongside the side chain of aromatic amino acids such as Tryptophan, while fucose residues tend to make contact with positive-charged amino acids including Arginine and Lysine [26]. Therefore, we hypothesized that Trp1721 and Lys1738 may be important for *N*-glycan-dependent intoxication. We therefore performed a mutagenesis study to evaluate the effect on intoxication of TcdA1^W14^ with W1721S and K1738S substitutions (S4D and S4E Fig). As shown in Fig 3E and 3F, both TcdA1^W14^-W1721S and TcdA1^W14^-K1738S substitutions reduce PTC3 intoxication effects. Moreover, the TcdA1^W14^-W1721S/K1738S double mutant showed much weaker effect on the membrane blebbing phenotype than that induced by the wild-type TcdA1^W14^, suggesting that both amino acid residues contribute to the binding of *N*-glycans. Taken together, these results confirm that the RBD-D of TcdA1^W14^ mediates the *N*-glycan-dependent intoxication, and identified two key amino acid residues that contribute to the interactions. This also suggests that the *N*-glycan binding mode of TcdA1^W14^ resembles that observed for C-type lectins.

### Sulfated glycosaminoglycan (sGAG) mediates intoxication by a TcdA2^TT01^-based toxin

**Fig 4:**
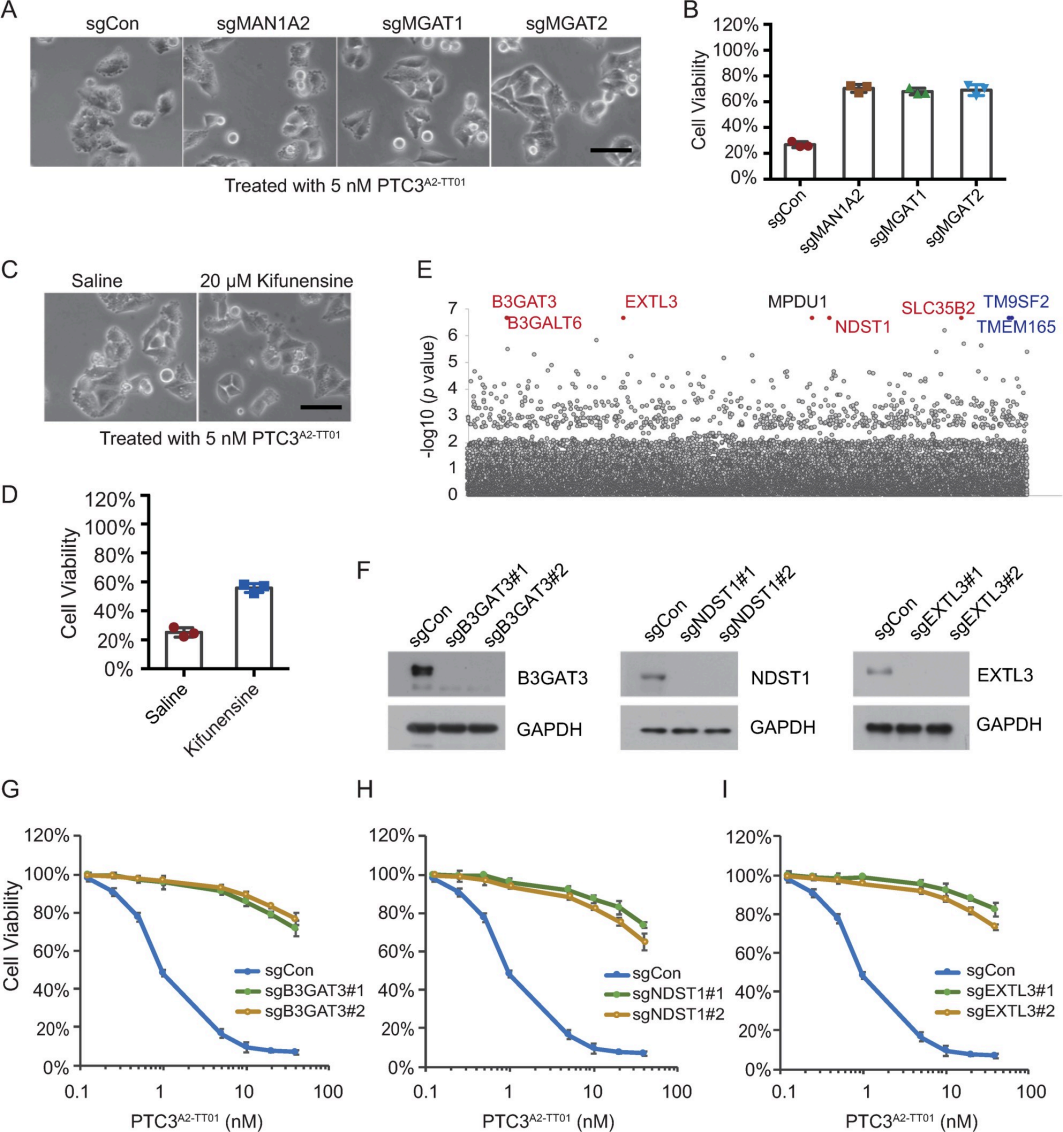
Sulfated glycosaminoglycan (sGAG) mediates the cell targeting of TcdA2. (A-B) Representative bright field micrographs of *N*-glycan-deficient and HeLa-Cas9-sgCon cells that were treated with 5 nM PTC3^A2-TT01^ (A). Cell viability was measured using CCK-8 assays (B). (C-D) HeLa-Cas9-sgCon cells pretreated with or without 20 μM Kifunensine for 12 h were exposed to 5 nM PTC3^A2-TT01^. Representative bright field micrographs (C) and the effects on cell viability (D) are shown. (E) Genes identified in the screens with PTC3^A2-TT01^ treatment were ranked based on the MAGeCK *p* values. Genes involved in sulfated glycosaminoglycan biosynthesis pathways are labeled in red. Two Golgi proteins, TMEM165 and TM9SF2, are labeled in blue. (F) The efficiency of gene knockout was determined by Western blot analysis with the indicated antibodies. GAPDH served as the loading control. (G-I) The indicated HeLa-Cas9 cells were pretreated with 20 μM Kifunensine for 12 h, and then exposed to indicated doses of PTC3^A2-TT01^. Cell viability was measured using CCK-8 assays (I) All error bars indicate mean ± SD. Each of the experiments was repeated three times.

Intriguingly, although the RBD-D of TcdA2^TT01^ can only weakly bind to *N*-glycans, a TcdA2^TT01^ based complex (PTC3^A2-TT01^) showed a robust effect on cell death, which was only partially blocked in *N*-glycan-deficient cells (Fig 4A and 4B). Similarly, kifunensine did not significantly protect cells against PTC3^A2-TT01^ (Fig 4C and 4D). Thus, we suspected that other mechanisms may be involved in the cellular recognition of TcdA2^TT01^. To explore the alternative pathway(s) involved in TcdA2^TT01^ recognition, we performed further genome-wide CRISPR-Cas9 screens. A lentiviral sgRNA library was transduced into HeLa-Cas9 cells as described in Fig 1C. To exclude any interference from potential *N*-glycan-mediated intoxication, HeLa cells were pre-treated with kifunensine before exposure to PTC3^A2-TT01^. After three rounds of selection with PTC3^A2-TT01^ (5, 10 and 20 nM, S5A Fig), the resistant population of HeLa-Cas9 cells was again analyzed using next-generation sequencing (NGS).

Very few genes involved in *N*-glycan synthesis were identified, with the top-ranked genes seen to encode key regulators involved in sulfated glycosaminoglycan (sGAG) biosynthesis pathways (Fig 4E and S1 and S3 Tables). These genes include enzymes involved in sulfated glycosaminoglycan (sGAG) synthesis, such as β-1,3-galactosyltransferase 6 (B3GALT6) and β-1,3-glucuronyltransferase 3 (B3GAT3), proteins that facilitate the sulfation of GAGs, such as *N*-deacetylase and *N*-sulfotransferase 1 (NDST1), and solute carrier family 35 member B2 (SLC35B2), as well as glycosyltransferases that are specifically required for the elongation of the heparan sulfate chain, such as exostosin like glycosyltransferase 3 (EXTL3). To validate this result, three representative sGAG-related genes, B3GAT3, NDST1, and EXTL3, were knocked-down in order to block the sGAG synthesis (Fig 4F). After preincubating with kifunensine, these sGAG-deficient cell lines showed increased resistance to the intoxication by PTC3^A2-TT01^, compared to the control cells (Fig 4G–4I). Taken together, these data demonstrated that in addition to *N*-glycans, sulfated glycosaminoglycans also mediate intoxication by TcdA2^TT01^-related Tc toxins, and corroborate the previous finding by Roderer *et al*. showing that diverse Tc toxins interact with heparin/heparan sulfates [21].

### The sulfate group of sulfated glycosaminoglycans is critical for mediating intoxication by TcdA2^TT01^-based toxin

**Fig 5:**
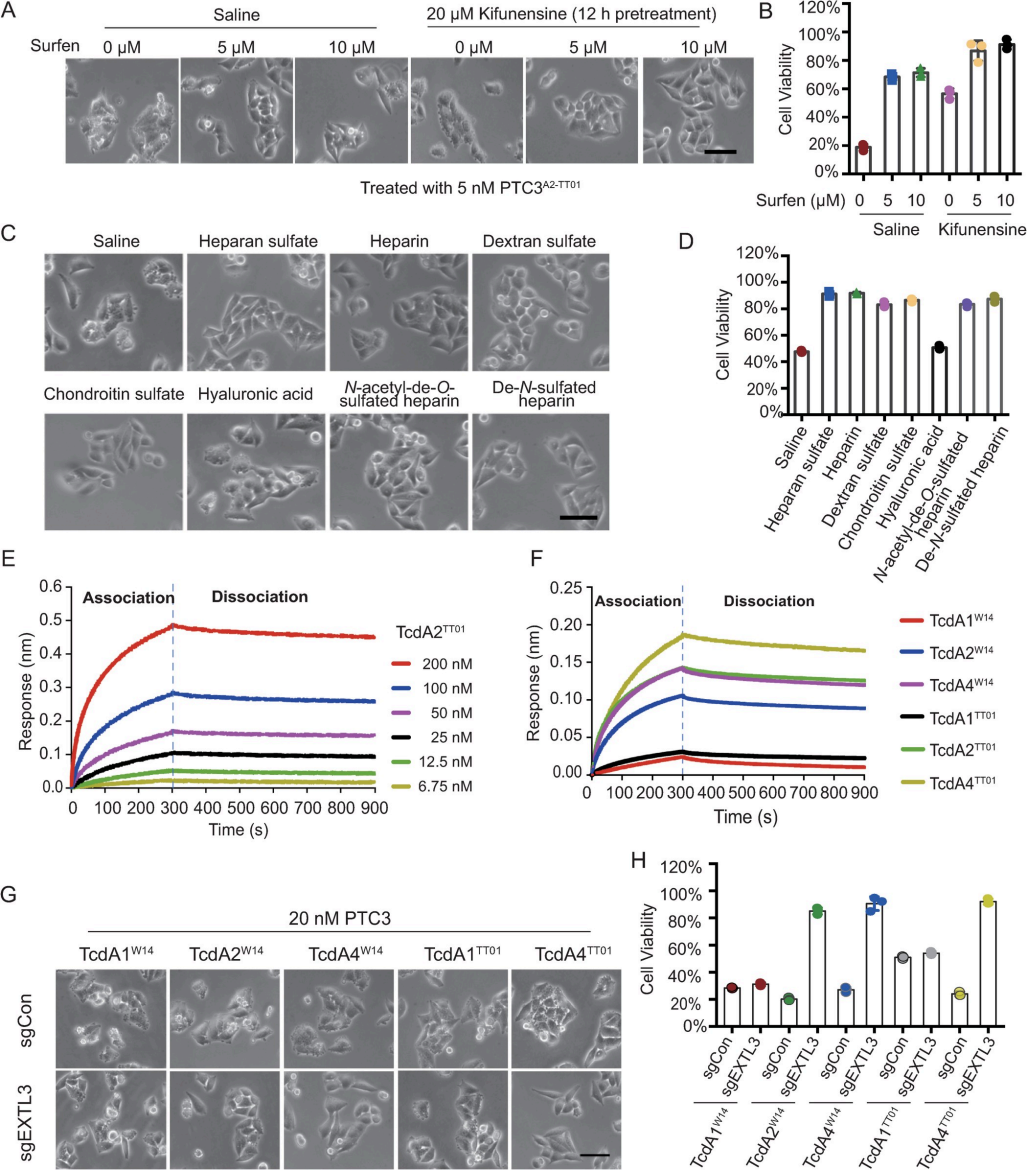
TcdA2^TT01^ binds with the sulfate group of sulfated glycosaminoglycans. (A-B) HeLa-Cas9-sgCon cells pretreated with or without 20 μM Kifunensine for 12 h were exposed to 0, 5, 10 μM Surfen, respectively. Representative bright field micrographs (A) and cell viability (B) are shown. (C-D) HeLa-Cas9-sgCon cells were pre-incubated with 20 μM Kifunensine for 12 h and the indicated glycosaminoglycans (all at 1 mg/ml) for 1 h, then treated with 5 nM PTC3^A2-TT01^. Representative bright field micrographs (C) and the effects on cell viability (D) are shown. (E-F) BLI sensorgrams of TcAs interacting with immobilized biotin-Heparin. (E) BLI sensorgrams of TcdA2^TT01^ interacting with biotin-Heparin. TcA pentamer concentrations were 6.75–200 nM. (F) The biosensors loaded with biotin-Heparin were exposed to 50 nM of the indicated TcAs, followed by washing in HBS-EP buffer. The association and dissociation phases of each group are separated by a dashed line. The Kinetic parameters were listed in S5C Fig. (G-H) Representative bright field micrographs of HeLa-Cas9-sgCon cells treated with indicated Tc toxins (G). Cell viability was measured using CCK-8 assays and shown in (H).

Glycosaminoglycans such as heparan sulfate have previously been implicated in microbial invasion of host cells [27]. For example, heparan sulfate not only functions a receptor/co-receptor for *H*. *pylori* VacA cytotoxin, but can also be recognized by *Clostridium difficile* toxin A, *via* the interaction with the charged sulfation group [28,29]. Roderer *et al*. also reported that both *Morganella morganii* TcdA4 and *Xenorhabdus nematophila* XptA1 interact with heparin/heparan sulfates [21]. To determine the mechanism underlying the sGAGs-mediated PTC3^A2-TT01^ intoxication, we investigated whether it relies on the electrostatic interactions between the sulfate group of sGAGs and TcdA2^TT01^. Surfen (bis-2-methyl-4-amino-quinolyl-6-carbamide) is a small molecule that can neutralize the negative charge of sGAGs. Pretreatment of HeLa cells with surfen significantly reduced the toxicity of PTC3^A2-TT01^ (Fig 5A and 5B), indicating that negative charges on sGAGs may be critical for the cellular recognition by TcdA2^TT01^.

Since a variety of sGAGs have been reported to mediate the interaction with toxins, we utilized a series of GAGs to investigate the role of sulfate groups in PTC3^A2-TT01^ intoxication. These competition assays showed that GAGs which possess the sulfate group, such as heparan sulfate, heparin, chondroitin sulfate and dextran sulfate, were all able to competitively inhibit the toxigenic effect of PTC3^A2-TT01^ in HeLa cells pretreated with kifunensine, while non-sulfated GAGs such as hyaluronic acid did not block PTC3^A2-TT01^ and so induced membrane blebbing (Fig 5C and 5D). Furthermore, we examined the effect of two different linkage types of sulfate groups. As shown in Fig 5C and 5D, both *N*-acetyl-de-*O*-sulfated heparin and de-*N*-sulfated heparin showed a similar protection against PTC3^A2-TT01^ intoxication. Taken together, these data demonstrate that sGAG can mediate PTC3^A2-TT01^ intoxication, which is dependent on the charged sulfate group.

### TcAs of different subfamilies may exhibit different target specificities

Recently, Roderer *et al*. also reported that TcAs of *Morganella morganii* and *Xenorhabdus nematophila* can strongly bind with heparins/heparan sulfates, compared with TcdA1^W14^ and TcaA-TcaB of *Yersinia pseudotuberculosis* [21]. Since TcdA2^TT01^ also exhibits sGAG-dependent cell binding, we next examined whether other *P*. *luminescens*-derived TcA proteins could also utilize sGAG to bind mammalian cells. To do so, we quantified the interaction between different TcAs and biotin-conjugated heparin by using biolayer interferometry (BLI). As shown in Fig 5E, the dissociation constants (K_D_) for TcdA2^TT01^-heparin is 2.77 nM (*k*_on_ of 8.44 ± 0.1 × 10^4^ M^−1^ s^−1^, and *k*_off_ of 2.34 ± 0.03 × 10^−4^ s^−1^). Other than TcdA2^TT01^, *P*. *luminescens* TcA homologues such as TcdA2^W14^, TcdA4^W14^, and TcdA4^TT01^, also showed strong binding with heparin (Figs 5F and S5B and S5C). In contrast, the binding signals for TcdA1^W14^ and TcdA1^TT01^ were much weaker, indicating that the cellular recognition of these two TcAs may not rely on an interaction with heparin. Consistently, knockout of EXTL3 significantly interfered with the action of Tc toxins assembled using TcdA2^W14^, TcdA4^W14^, or TcdA4^TT01^, while only marginally effecting intoxication by those using TcdA1^W14^- and TcdA1^TT01^ subunits (Fig 5G and 5H and S2B and S5D). Taken together, these data suggest that the binding of TcAs with sGAG is a more representative cellular recognition mode for *P*. *luminescens* Tc toxins.

**Fig 6:**
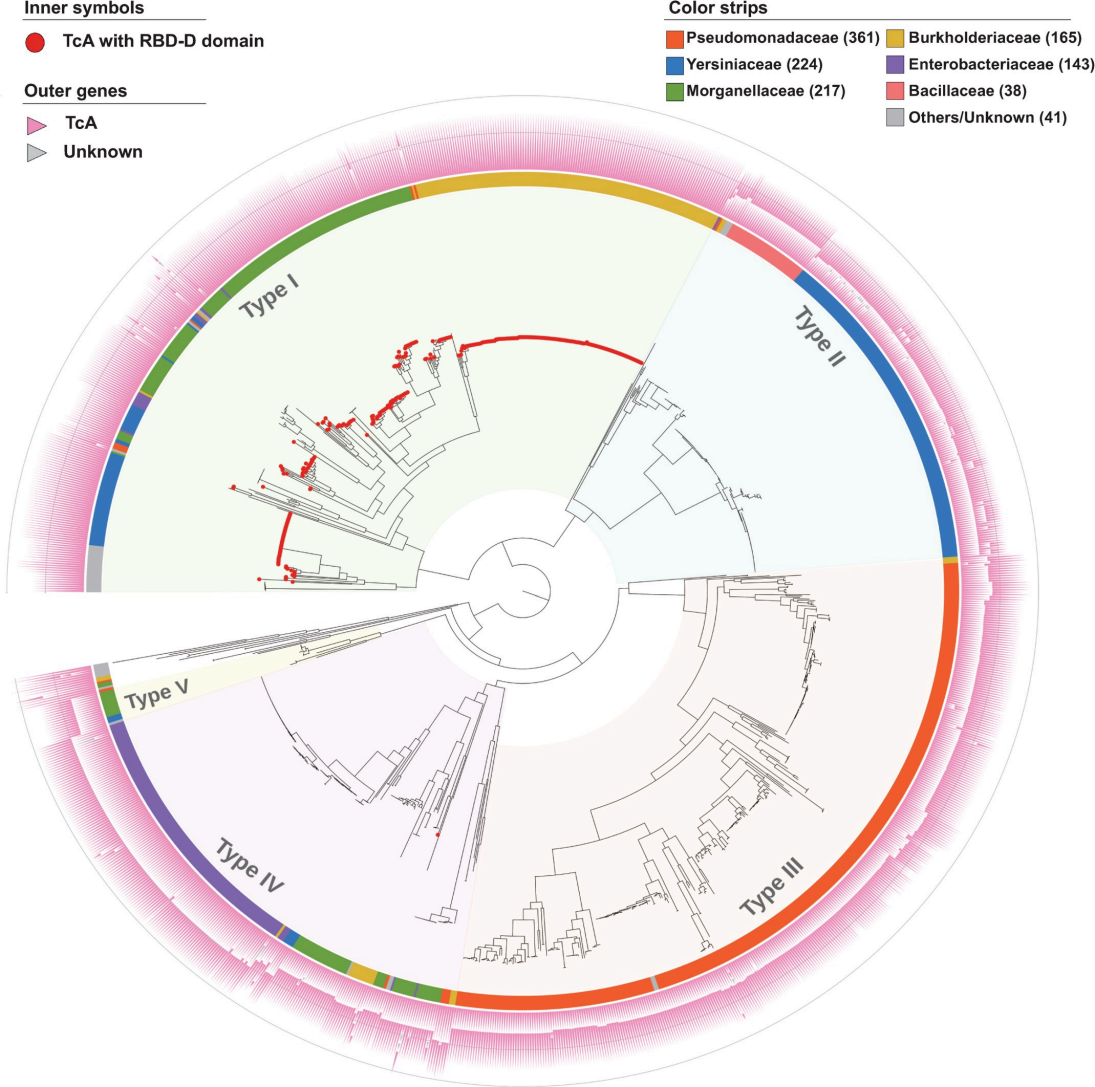
The taxonomic distribution of 1,189 predicted TcC proteins. Maximum-likelihood tree of 1,189 predicted set of TcA proteins (root on midpoint), based on the concatenated multiple alignment sequences of the three known domains, including VRP1 (PF03538), Neuraminidase (PF18413) and TcA_TcB_BD (PF18276). The tree was constructed by FastTree under WAG models with gamma optimization. TcA proteins encoding the RBD-D domain (PF18518) were indicated by solid red circles at the leaf. Outside strips were color coded by bacterial families as indicated by the key. Outer triangles represent the genetic organization of each TcA set. The five distinct types of TcA group are highlighted with colored shadows for clarity. The tree scale represents substitutions per site. A detailed rectangular tree is given in S7 Fig.

To further explore the potential binding modes of TcAs from other species, we performed a genome-wide analysis of TcA homologues across multiple bacterial species. Typically, TcAs from *P*. *luminescens* represent a single protein that harbors at least three conserved domains, named the N-terminal VRP1 domain (PF03538), the middle Neuraminidase domain (PF18413) and the C-terminal TcA_TcB_BD domain (PF18276) (S6 Fig). Nevertheless, there are functional examples of “split” TcA homologues, such as TccA1 and TccB1 of *P*. *laumondii*, and TcaA and TcaB in *Y*. *pseudotuberculosis* (S6 Fig). Based on this information, we have curated a collection of 1,189 putative TcA homologues from 1,039 bacterial genomes derived from our open access dbTC database (http://www.mgc.ac.cn/dbTC/). For this set, a valid “TcA” refers to a either a single protein or 2–3 consecutive proteins that together encode(s) all of the three aforementioned conserved domains in the appropriate order. Then, based on a concatenation of the three conserved domains, we constructed a maximum-likelihood phylogenetic tree of the 1,189 predicted TcA protein set (Fig 6).

The majority (98.8%) of identified TcAs fall into five distinct phylogenetic clades, that we designate types I to V, with only 14 outliners which do not fit this classification. Perhaps unsurprisingly, these TcA subfamilies show a good correlation with both genetic organization of the TcA protein(s) and taxonomic origin of the genomes. Type I TcA is the largest and the most variable group, containing genomes from several bacterial species, including *Burkholderia*, *Photorhabdus*, and *Xenorhabdus*. Most members of the type I subfamily are single large proteins that most closely resemble those reported in *P*. *luminescens*. Of note, though RBD-D, which mediates binding with *N*-glycans is specifically required for TcdA1^W14^, 83.4% (331/397) of the TcA proteins from type I encode the RBD-D (PF18518) (S7 Fig), suggesting that this domain may exhibit more functions other than *N*-glycans binding.

TcA homologues from all of the other subfamilies generally consist of two different open reading frames encoded in immediate proximity to one-another. Nevertheless, the genetic organization and the total molecular weight of TcAs are clearly different between the types II to V subfamilies (Fig 6). The type II TcAs are highly represented in *Yersinia* genomes, comprising separate ~90 kDa A-subunits and ~130 kDa B-subunits. Type III TcAs were found to be predominately distributed in genomes from the genus *Pseudomonas*, with subunit molecular weights of ~100 kDa and ~150 kDa, respectively. The type IV TcAs, which are prevalent in *Salmonella*, comprise of the largest A and B subunits, which are ~180 kDa and ~160 kDa, respectively. Though very little is known about the cellular recognition mechanisms of these TcAs, the differences in both A and B subunits suggests that the TcAs of these three subfamilies might exhibit distinct receptor specificities.

The *Y*. *entomophaga* MH96 TcA subunit proteins YenA and YenB, were categorized into the type V TcA subfamily. Previous reports demonstrated that these TcAs were decorated by two endochitinases (Chi1 and Chi2), which are critical in allowing the toxin complex to overcome the chitin-based barrier immunity seen in the guts of insects [20]. Intriguingly, although this type V subfamily consists of only 16 members (S7 Fig), most of them also harbor chitinase gene(s) immediate downstream of TcA genes, suggesting Tc toxins derived from this type V subfamily may represent potent insecticidal candidates for biological control of plant diseases. Taken together, these results supported the reliability of our classification of the TcA subfamilies, and further implied that each type of TcA may represent specific receptor-binding features given the distinct genetic organizations.

## Discussion

Cellular recognition by TcA subunits is a prerequisite for the activity of Tc toxins, and appears to determine host cell specificity. To take an unbiased approach towards identifying mammalian cell targeting mechanisms of different TcAs, we utilized a genome-wide CRISPR library screening strategy. This approached relied on the ability of the toxin complex to kill target cells that continued to express the critical factors that mediate the toxin action, but not to intoxicate those in which these factors were ablated. The intoxication phenotype in HeLa cells is a seen as Tc toxin-induced membrane blebbing and cell death. In this study we have demonstrated two distinct pathways that are required for the action of certain Tc toxins, involving the synthesis of either *N*-glycans or sGAGs.

In addition, using a bioinformatic analysis, we have identified 1,189 TcA protein homologues from 1,039 bacterial genomes. According to similarities in gene structure and sequence characteristics, we have classified these TcAs into five distinct subfamilies, which we propose likely employ class and species-specific target cell recognition mechanisms. There are several well described examples of bacterial exotoxins that use cell surface glycans as receptors [30]. One of the best-characterized example is the AB_5_ cholera toxin, produced by *Vibrio cholera*, which consists of one A subunit and five B subunits [31]. The homopentamer formed by the B subunits binds to the Galβ1-3GalNAc moiety of the GM1 ganglioside, a cell surface glycan receptor, *via* the C-terminal repetitive domain (CRD) located on the base of the cholera toxin [32]. Moreover, Yamaji *et al*. previously performed a genome-wide screening for the receptor of the subtilase cytotoxin (SubAB), another AB_5_ toxin [33]. They reported that knockout of MGAT1 can block the toxic effect of SubAB, suggesting that *N*-glycans also serve as SubAB receptors. Similar to the AB_5_ toxins, the TcA pentamer that forms the receptor binding component for the Tc toxins, can also bind *N*-glycans and facilitate delivery of the TcC toxic domain into the target cell [20,21]. Of note, the molecular species of N-glycan could be vary between different hosts. For example, HepG2 has been reported to express more sialylated glycans than HeLa cells, while MCF-7 cells express less glycans [34]. Our data also showed that the difference between the tested mammalian cell lines in Tc toxin sensitivity, suggests that a further characterization of *N*-glycans species in toxin-related studies may provide more information about the host tropism of various pathogens.

TcdA1 can bind to BSA-Lewis X glycoconjugate *via* the RBD-D domain, with a dissociation constant (KD) of 5.8±0.7μM [21], suggesting that the RBD-D domain may contribute to the diverse pattern of host tropism. We therefore performed a sequence alignment of the RBD-D from all 332 RBD-D-containing TcAs (331 in type I superfamily and 1 in Type IV superfamily). Unfortunately, few similarities could be found within this domain (S8 Fig). This is in line with our results that showing that TcdA1^TT01^, a RBD-D-containing TcA, can function normally in both wild-type and *N*-glycan-deficient cells. In addition, Piper *et al*. demonstrated that YenTc, which does not contain a RBD-D domain, can also bind to some classes of glycans [20]. These data suggest that the *N*-glycan-binding role of RBD-D cannot be applied to all RBD-D-containing TcAs. We further generated an evolution-similarity matrix based on the sequences of 332 RBD-D-containing TcAs. As shown in S9 Fig, the RBD-D similarity showed a good correlation with the taxonomic origin of the genomes, which differed from species to species, suggesting that the RBD-D domains of different TcAs may interact with different host factors. Of note, a putative TcA from the human pathogenic strain *Photorhabdus asymbiotica* (TcdA^PA^, WP_012777086) harbors a RBD-D that shares 83% and 81% amino acid identities with those of TcdA1^W14^ and TcdA2^TT01^, respectively. Considering that the Tc toxin may contribute to the pathogenesis of *P*. *asymbiotica*, it is worth considering if blockade of the *N*-glycan-binding of TcdA^PA^ could control *P*. *asymbiotica* infection. Taken together, these observations indicate that N-glycans can act as receptors for various toxins employing a pentameric recognition and delivery complex. It also suggests that multi-valent synthetic *N*-glycan ligand/analogues could be used as protective agents against Tc toxin mediated infections. Of note, other than RBD-D, several TcAs such as TcdA1^W14^, contain three other potential receptor-binding domains (RBD-A, RBD-B, and RBD-C) as well as a neuraminidase-like domain, which may also contribute to the cellular recognition of Tc toxins [11]. For example, the heparin binding site of Xn-XptA1 is located in a cleft formed by RBD-B, RBD-D and the neuraminidase-like domain [21]. Piper *et al*. also indicated that the neuraminidase-like domain of YenTcA harbors lectin activity, and may bind with cell surface glycans [20]. Thus, these domains may provide alternative mechanisms for the binding of diverse Tc toxins with host cells, although further analysis will be needed to screen the putative ligands that mediate the interactions.

Glycosaminoglycans (GAGs) are complex linear polysaccharides that are ubiquitously expressed at the cell surface, and exploited by a variety of bacterial toxins as essential host cell receptors required for intoxication [35,36]. For example, Heparan sulfate, one type of GAGs, has been reported as a critical host factor that mediates the delivery and translocation of *Yersinia* outer proteins (Yops) [37]. In addition, Chondroitin sulfate is suggested to play an important role in the toxicity of anthrax toxin, produced by *Bacillus anthracis* [38]. Both our findings (Fig 5F) and those of Roderer *et al*. demonstrate that a series of TcAs can bind with sulfated glycosaminoglycans (sGAG), such as heparin [21]. However, the sGAG-binding pocket is not conserved in the *Morganella morganii* TcdA4 (located in the α-helical domain of the shell) or in the *Xenorhabdus nematophila* XptA1 (which is located in a gap between the neuraminidase-like domain, RBD B and RBD D). Moreover, although *Clostridium difficile* toxin A also utilizes sGAGs as host cell receptors, the exact binding sites for sGAGs remains to be determined. Therefore, at this point we cannot reliably determine the potential sGAG-binding region to facilitate the further analysis. In addition, Roderer *et al*. reported that the binding signals of TcdA1^W14^ was weaker than the values of *Morganella morganii* TcdA4 for porcine intestinal mucosa-derived heparin [21], which is in line with our BLI results (Fig 5F). Together with the observation that EXTL3 knockout in HeLa cells showed marginal effects on the action of PTC3^W14^, it suggested that sGAGs may not be required for the mammalian cell targeting of TcdA1^W14^. Although the interaction between TcdA1^W14^ and natural heparin mixtures is weak, TcdA1^W14^ can indeed bind to several specific types of heparin oligosaccharides with relatively high affinities [21]. Whether these interactions is required for the insect cell recognition of TcdA1^W14^ needs to be investigated.

Intriguingly, a study by Tao *et al*., that also used a CRISPR known-down screening strategy, recently reported that sulfated glycosaminoglycans act as host cell receptors of the *Clostridium difficile* toxin A [28]. The top-ranked genes shown to contribute to the action of *C*. *difficile* toxin A are almost identical to those we enriched for in our TcdA2^TT01^ CRISPR screen. This indicates that the *C*. *difficile* toxin A and TcdA2^TT01^-related Tc homologues share a similar cellular recognition mechanism. Moreover, this study showed that blocking sGAG binding by GM-1111, a sulfated heparan sulfate analogue, can also reduce the toxicity of *C*. *difficile* toxin A in the colon [28]. Thus, blockade of GAG-binding by engineered GAG mimetics, sulfated compounds, as well as GAG-digesting enzymes, could represent promising antimicrobial strategies for the development of therapeutics for infectious diseases facilitated by toxins such as Tc toxin.

In addition, two golgi-linked genes TM9SF2 and TMEM165 were identified in both *C*. *difficile* toxin A and the TcdA2^TT01^ Tc toxin screening (Fig 4E), suggesting these two genes may be involved in the synthesis of sulfated glycosaminoglycans. TM9SF2 was previously found to regulate the localization of NDST1, a Golgi complex-localized enzyme that catalyzes *N*-sulfation of heparan sulfate, and is therefore critical for chikungunya virus infection [39]. Besides these screening experiments, three independent groups have also suggested that TM9SF2 plays a conserved role in Shiga toxin-induced cell death [40–42]. Tian *et al*. demonstrated that TM9SF2 not only regulates glycosylation in Golgi, but also modulates endosomal trafficking in general [42]. These observations suggest that TM9SF2 might be a potential target for treating various infections. Besides TM9SF2, TMEM165, a Mn^2+^ transporter, has also been identified as one critical host factor that mediates the intoxication of Shiga toxin and ricin [41,42]. Intriguingly, knockdown of TMEM165 in zebrafish showed a defect in chondroitin sulfate proteoglycan expression by alterations in either core protein expression or glycosaminoglycan chain composition [43]. Thus, TMEM165 inhibition may effect discrete aspects of GAG synthesis, although further analysis will be required to demonstrate the precise mechanism.

Although the Tc toxins were first identified almost two decades ago, current knowledge regarding Tc toxin biology has mainly come from structural investigations that focus on the complex assembly and mechanisms of delivery such as the TcC autocleavage and injection [9,10,13,14,16], while less attention has been paid to the host cell recognition by Tc toxins. Recently, two independent studies utilized comprehensive glycan microarray screens to identify potential receptor ligands for Tc toxins, and found that *N*-glycans and heparan sulfates can bind with several TcAs [20,21]. Genome-wide CRISPR/Cas9 screening has emerged as a powerful genome-editing technology for studying the intoxication of mammalian cells by toxins. Therefore, we decided to utilize this strategy to explore the host factors that are required for the actions of Tc toxin homologues. Of note, both our results and those of Roderer *et al*. demonstrated the interaction of TcdA1^W14^ with *N*-glycans. Since we employed a different, and unbiased, strategy to that study (CRISPR knock-down as opposed to glycan array screens), it is reassuring to see the two studies giving consistent findings, indicating that the genome-wide CRISPR/Cas9 screening would be a promising strategy to identify host factors for other Tc toxins derived from distinct subfamilies indicated in Figs 6 and S7.

It should be noted that certain other bacterial toxin host recognition systems have been shown to use binding to multiple receptors to facilitate efficient the cell entry and internalization. Other than glycans and glycosaminoglycans, several cell surface proteins such as CD31 and integrins have also been shown to contribute to the target specificity of distinct toxins [44,45]. Thus, it remains an open question as to whether or not Tc toxin binding also involves receptors other than glycans and glycosaminoglycans. Interestingly, our current study did not identify enrichments for any specific cell surface proteins. Nevertheless, while it is beyond the scope of the current study, we cannot rule out that further “deeper” screens might yet provide indications of additional receptors for Tc toxins.

Since the intoxication has been observed for several mammalian cell types, the Tc toxins have been proposed as a potential virulence factors that may contribute to diverse human infectious diseases. However, the exact roles of Tc toxin homologues in the majority of bacterial infections remains unclear. This is in part a consequence of a lack of systematic study of the distribution of Tc toxins in the different pathogenic bacteria. Here we have addressed this issue by performing a comprehensive analysis of TcA homologues in bacterial genomes available in public databases, and the construction and provision of an open access database. In total, a set of 1,189 TcA protein homologues were identified from 1,039 bacterial genomes which we classified into 5 distinct subfamilies. Notably, several TcAs derived from the human pathogenic bacteria *Yersinia* and *Salmonella* fell into two different subfamilies, suggesting these bacteria may utilize Tc toxins to facilitate their invasion and survival in host cells. Further detailed studies regarding these human pathogenic bacteria-derived Tc toxins will no doubt contribute to our understanding about this polymorphic toxin system.

Appropriate host cell recognition by toxins is often critical for successful infection strategies used by pathogenic bacteria. Using a CRISPR-Cas9-based sgRNA knock-down library, we have performed a genome-wide loss-of-function screening to identify the critical host cell enzymic processes required for the recognition and action of several Tc toxins. We have demonstrated that not only *N*-glycans but also glycosaminoglycans can serve as receptors for different TcA pentamers. Taken together with our bioinformatic study characterizing the diversity of Tc homologues among different bacterial species, we provide valuable information for elucidation of the pathogenic roles of Tc toxins. Future research focusing on the structural basis of the toxin-receptor interaction and the design of protective compounds could not only create effective therapies for patients with Tc toxin-facilitated diseases, but also expand our understanding of this versatile bacterial weapon. Finally, these findings will inform on selection strategies for potential biotechnology tools for new crop-protection agents and biomedical applications.

## Materials and methods

### Cell culture

HEK293T cell (CRL-11268, RRID:CVCL_1926), MEF cell (CRL-2991, RRID:CVCL_L690), Vero Cell (CCL-81, RRID:CVCL_0059), L929 cell (CRL-6364, RRID:CVCL_0462) were obtained from ATCC and cultured according to the manufacturer’s protocol. HeLa cell (CCL-2, RRID:CVCL_0030) was obtained from ATCC and cultured in Dulbecco’s modified Eagle’s medium (DMEM, Cat# 11965092, Thermo Fisher Scientific) supplemented with 10% FBS (Cat# FS101-02, TransGen Biotech), 100 U/mL Penicillin and 100 μg/ml Streptomycin (Cat# 15140148, Thermo Fisher Scientific) at 37°C under 5% CO_2_. HeLa cells stably expressing Cas9 (HeLa-Cas9) were generated by transducting Lenti-Cas9-Blast virus (#52962, Addgene, RRID:Addgene_52962), and selecting using 10 μg/mL Blasticidin S (Cat# A1113903, Thermo Fisher Scientific).

### Plasmids

The coding sequences of TcdA1^W14^, TcdA2^W14^, TcdA4^W14^, TcdB2, TccC3 were cloned from the genome of *Photorhabdus luminescens W14* (lab stock). TcdA1^TT01^, TcdA2^TT01^, and TcdA4^TT01^ were cloned from the genome of *P*. *luminescens TT01* (lab stock). All TcAs was cloned into RSFDuet-1 (Novagen) with N-terminal 6×His tag. TcdB2 and TccC3 fusion protein was constructed incorporating a 5-aa linker region encoding Arg-Gly-Ser-Arg-Pro, and cloned into pETDeut-1 (Novagen) with N-terminal 6×His tag and C-terminal Flag tag. To engineer the mutants of indicated TcAs, site-directed mutagenesis or seamless cloning were performed according to the manufacturer’s protocol of pEASY-uni seamless cloning and assembly Kit (Cat#CU101-01, TransGen Biotech). All constructs were confirmed by DNA sequencing (Sangon Biotech, Shanghai).

### Tc toxin purification

All Tc toxin components were prepared according to the previous protocol with some modifications [15]. His-tagged TcA proteins were expressed in *E*. *coli* BL21 (DE3) cells (Cat# CD601-02, TransGen Biotech). Expression was performed in 1L LB medium with 50 mM IPTG induction for 24 h at 20°C, with aeration by 250 rpm shaking. The cells were then collected and sonicated. Cell lysates were centrifugated and then applied to the Ni-NTA affinity column (Cat# 30250, Qiagen). The elution fraction was further purified using gel filtration (Superose 6 Increase 10/300 GL, GE Healthcare) using an AKTA avant25. The TcB-TcC expressing plasmids were transduced into BL21-CodonPlus (DE3)-RIPL cells (Cat# 230250, Agilent Technologies). A single clone was inoculated into 1L LB medium containing 25 μM IPTG, with aeration by 250 rpm shaking for 4 h at 28°C, followed by 48 h shanking at 20°C. The cells were harvested by centrifugation and lysed by ultrasonication. His-tagged TcB-TcC fusion protein was purified by anion exchange chromatography, followed by a Ni-NTA affinity column and final gel filtration as described above. All TcAs and TcB-TcC fusion proteins were concentrated to about 1 mg/ml, using Amicon filter devices (Millipore).

For the purification of the holotoxin complex formed by indicated TcA and TcdB3-TccC3 fusion proteins, TcA and TcdB2-TccC3 were mixed in a 2:1 mole ratio with a buffer containing 50 mM Tris pH 8.0, 100 mM NaCl, and 5% glycerol. The mixture was then applied to gel filtration using a Superose-6 10/300 GL column (Cat# 17-5172-01, GE Healthcare). The elution fractions between 13–14 ml were concentrated and finally used for cell intoxication assay.

### Western blot

In brief, samples were subjected to SDS-PAGE and then transferred onto PVDF membranes. Membranes was incubated with indicated primary antibodies and corresponding secondary HRP antibodies, and examined by using enhanced chemoluminescent detection reagent (Thermo Fisher Scientific). Mouse molyclonal anti-FLAG M2 antibody (Cat# F3165, RRID: AB_259529) was purchased from Sigma. Rabbit polyclonal anti-MAN1A2 antibody (PA5-56974, RRID: AB_2643718) was purchased from Thermo Fisher Scientific. Rabbit polyclonal anti-NDST1 antibody (26203-1-AP) was purchased from Proteintech. Mouse molyclonal anti-EXTL3 antibody (sc-271986, RRID:AB_10709180) and anti-B3GAT3 antibody (sc-390475) were purchased from Santa cruz. Rabbit monoclonal anti-MGAT1 antibody (ab180578, RRID:AB_2800510) and anti-MGAT2 antibody (ab184965) were purchased from Abcam.

### Negative stain electron microscopy

After gel filtration, 4 μl sample droplets were applied on freshly glow-discharged copper grids (G2400C, Agar scientific) covered by a thin, continuous carbon film. The samples were left for 20 s on the grid before blotting with filter paper, and stained with 0.04% uranyl acetate twice, air-dried for 20 s. All images were taken with a JEOL JEM-1400 electron microscope equipped with a LaB6 cathode operated at 120 kV. Digital electron micrographs were recorded with a 4k × 4k CMOS camera F416 (TVIPS) using minimal dose conditions.

### Cell intoxication

HeLa cells were seeded into 24-well plate (1 × 10^5^ per well), and grown adherently on sterile coverslips overnight in 500 μL culture medium. The indicated holotoxin was subsequently added. For blockade of the biosynthesis of *N*-linked glycans, HeLa cells were pretreated with 20 μM kifunensine for 12 h before Tc toxin stimulation. For F-actin staining, the cells were fixed with 4% paraformaldehyde and then permeabilized with PBS containing 0.1% Triton X-100 for 15 min. Coverslips were then incubated with Alexa Fluor 568 Phalloidin (Cat# A12380, Thermo Fisher Scientific) for 30 min and washed for 3 times with PBS. Coverslips were mounted with ProLong Gold Antifade Mountant with DAPI (Cat# P36941, Thermo Fisher Scientific). All the images were captured by EVOS FL Auto 2 Imaging System (Thermo Fisher Scientific).

### Cell viability assay

HeLa cells were seeded in 96-well microplates and incubated until 60% confluence, then exposed to the indicated doses of Tc toxins. The cells were further incubated for 24 h in toxin-containing medium and then 10 μL CCK-8 solution and incubated for 2 h. The cell viability was measured by Varioskan flash spectral scanning multimode reader (Thermo Scientific) at wavelength of 450 nm. Eight hour post stimulation, bright field microscopy was performed with Leica DMi1 Microsystems equipped with an ICC50 W/E digital camera, and captured by using the Leica LAS EZ software (Leica).

### CRISPR-Cas9 screen

Lentiviral sgRNA plasmid libraries were generated using the human GeCKO-V2 sgRNA library (Addgene, #1000000049) consists of 123,411 sgRNAs targeting 19,050 protein-coding genes (six sgRNAs per gene) and 1,864 microRNAs (four sgRNAs per microRNA). The titer of lentiviral sgRNA libraries was measured as colony-forming units (CFU) per mL. HeLa-Cas9 cells (2 × 10^7^) were transduced with a multiplicity of infection of 0.2 to provide 100 × coverage of each gudie RNA. Two days post infection, cells infected by lentivirus were further selected by puromycin. For the first round of screening, the cell library was exposed to 5 nM PTC3 for 24 h. The survival cells were seeded and cultured without PTC3 until about 80% confluence. Cells were then subjected to the next two round of selection (10 and 20 nM PTC3). Genomic DNA from 2 × 10^7^ of the HeLa cells (unselected control and selected with PTC3) was isolated (Blood & Cell Culture DNA Mini Kit, Cat#13323, Qiagen). Three biological replicates were performed for each selection. The genomic DNAs of three replicates were pooled together, and then amplified by PCR with primers lentiGP-1_F (AATGGAC TATCATATGCTTACCGTAACTTGAAAGTATTTCG) and lentiGP-3_R (ATGAATACTG CCATTTGTCTCAAGATCTAGTTACGC). Next-generation sequencing was performed by a commercial vendor (OBiO technology). Analysis of enriched guides was performed using MAGeCK analysis. The sgRNAs are ranked based on *P*-values calculated from the NB model. A modified robust ranking aggregation (RRA) algorithm named α-RRA is utilized to identify positively or negatively selected genes. If one gene has no effect on selection, the sgRNAs targeting this gene should be uniformly distributed across the ranked list of all the sgRNAs [46].

### Model building

Structural modeling was performed using SwissModel. For both TcdA1 and TcdA2 of *P*. *luminescens* TT01, the structure of TcdA1 of *P*. *luminescens* W14 (PDB: 4O9Y) was used as a template. Figures were prepared using PyMOL.

### Biolayer interferometry

Biolayer interferometry (BLI) assays were performed on the Octet Red Instrument (fortéBIO). Biotin conjugated heparin from porcine intestinal mucosa (5 μg/ml, B9806, Sigma) was immobilized onto streptavidin biosensors (fortéBIO) and balanced with HBS-EP buffer (10 mM HEPES, pH 7.4, 150 mM NaCl, 3.0 mM EDTA, and 0.005% Tween-20). The biosensors were then exposed to indicated doses of TcdA2^TT01^, or 50 nM of the indicated TcAs, followed by washing in HBS-EP buffer. BLI sensorgrams were measured with 300 s association and 600 s dissociation time, and corrected for background association on mock biosensors. The binding affinities between TcAs and heparin were monitored using the Blitz system (ForteBio). For calculating the dissociation constant (KD) of the TcdA2^TT01^-heparin binding, a global fit according to a 1:1 binding model was utilized.

### Phylogenetic analysis of TcA proteins

A total of 2,315 TcA related genes available from the dbTC database (http://www.mgc.ac.cn/dbTC/) were used to identify known protein domains by batch CD-Search of the conserved domain database with default parameters [47]. Though TcA in *P*. *luminescens* W14 is a single protein that encodes at least three conserved domains, including the N-terminal VRP1 (PF03538), the middle neuraminidase (PF18413) and the C-terminal TcA_TcB_BD (PF18276), the split homologues in *Yersinia* were confirmed to be functional previously [17,20]. Therefore, extensive manual curation was conducted based on the criterion that a valid set of *tcA* gene(s) may encode a single protein or 2–3 consecutive proteins, but all of the three aforementioned domains should present in a logical order. The protein sequences of VRP1, Neuraminidase and TcA_TcB_BD domains of each valid TcA set were extracted by custom Perl script based on the CD-Search results, and then fed to the Clustal-Omega program to generate multiple sequence alignment (MSA) of each domain individually. The three MSA files were concatenated accordingly to form a single MSA before the FastTree program was employed to construct the maximum-likelihood phylogenetic tree under Whelan Goldman (WAG) models with gamma optimization [48]. The iTOL online server was used to annotate and visualize the phylogeny [49].

### Statistical analysis

Unpaired Student’s t tests were performed using Excel software (Microsoft) or GraphPad Prism 6 (http://www.graphpad.com).

## Supporting information

S1 Table The sgRNA counts from control library cells and toxin-resistant cells. (XLSX)

S2 Table Identification of sgRNAs enriched in PTC3-W14 screening by MAGeCK analysis. (XLS)

S3 Table Identification of sgRNAs enriched in PTC3-A2-TT01 screening by MAGeCK analysis. (XLS)

S1 Fig The preparation of PTC3 and the effect of *O*-linked Glycans on the cellular recognition of the TcdA1^W14^ pentamer. (A-B) The *P*. *luminescens* W14*-*derived TcdA1 and TcdB2-TccC3 fusion proteins were co-incubated at 4°C overnight, and then subjected to gel filtration analysis (A). The indicated fractions were analyzed by SDS-PAGE(B). (C) Negative stain electron micrographs of PTC3^W14^ (Fraction #14). Scale bars: 100 nm. (D) HeLa, HeLa-Cas9 and HeLa-Cas9-sgCon cells were treated with indicated doses of PTC3^W14^ for 24 h. Cell viability was measured using CCK-8 assays. (E-F) C1GalT11-KO and control HeLa-Cas9-sgCon cells (sgCon) were exposed to 20 nM PTC3^W14^. Representative bright field micrographs (D) and the effects on cell viability (E) were shown.
(TIF)

S2 Fig PTC3 assembled by TcdA1^TT01^ was less potent than PTC3^W14^ on HeLa cell targeting. (A) Homology model of TcdA1^TT01^. Swiss model program was used to construct a homology model of TcdA1^TT01^ by alignment with TcdA1^W14^ (PDB: 4O9Y). The RBD-D of TcdA1^TT01^ were indicated in pink. (B) Coomassie blue staining and Western blot analysis of indicated Tc toxins. All these Tc toxins are prepared by co-incubation of TcdB2-TccC3-Flag fusion protein with indicated TcAs. The autocleaved TcC C-terminal fractions (TccC3-CTR) were detected by the Flag antibody. (C) HeLa-Cas9-sgCon cells were treated with 20 nM PTC3^TT01^ or PTC3^W14^ for 8 h. Cells were fixed, permeabilized and stained with with Alexa 568-phalloidin (red) and DAPI (blue). Representative fluorescence and bright field micrographs were shown. Scale bars, 5 μm. (D-E) HeLa-Cas9-sgCon cells were pretreated with saline or 20 μM Kifunensine for 12 h, then exposed to 20 nM PTC3^TT01^. Representative bright field micrographs were shown (D). Cell viability was measured using CCK-8 assays (E).
(TIF)

S3 Fig The RBD-D domain of TcdA1^W14^ is required for the *N*-glycan-mediated intoxication of Tc toxins. (A-D) Tc toxins formed by indicated TcAs and TcdB2-TccC3 were subjected to gel filtration analysis (A and C). Negative stain electron micrographs of the purified toxins (Fraction #14) were shown (B and D). Scale bars: 50 nm. (E-F) HeLa-Cas9-sgCon cells were pretreated with saline or 20 μM Kifunensine for 12 h, then exposed to indicated Tc toxins. Representative bright field micrographs were shown (E). Cell viability was measured using CCK-8 assays (F).
(TIF)

S4 Fig Sequence alignment of the RBD-D domain and the assembly of Tc toxins formed by TcdA1^W14^-W1721S/K1738S and TcdB2-TccC3. (A) Sequence alignment of the RBD-D domains derived from TcdA2^TT01^ or TcdA1^W14^, colored from minimum (white) to maximum (dark grey) conservation. The two sites that affect the binding to *N*-Glycans are indicated with red triangles. (B-C) HeLa-Cas9-sgCon cells were pretreated with saline or 20 μM Kifunensine for 12 h, then exposed to indicated Tc toxins. Representative bright field micrographs were shown (B). Cell viability was measured using CCK-8 assays (C). (D) Tc toxins formed by TcdA1 mutants (TcdA1^W14^-W1721S, TcdA1^W14^-K1738S and TcdA1^W14^-W1721S/K1738S) and TcdB2-TccC3 were subjected to gel filtration analysis. (E) Negative stain electron micrographs of the purified toxins (Fraction #14, as shown in D). Scale bars: 50 nm.
(TIF)

S5 Fig Schematic drawing of the screening of host factors that are required for the intoxication of PTC3^A2-TT01^ and the preparation of Tc toxins assembled by TcdA2^W14^, TcdA4^W14^, and TcdA4^TT01^. (A) Schematic drawing of the screening of PTC3^A2-TT01^ receptors. HeLa cells stably expressing Cas9 (HeLa-Cas9) were transduced with lentiviral GeCKO v.2 sgRNA libraries. These cells were pretreated with 20 μM Kifunensine for at least 12 h, and then exposed to increased doses of PTC3^A2-TT01^ (5, 10 and 20 nM). Cells that were not treated with PTC3 were served as controls. Total genomic DNA from 2×10^7^ selected and control cells was used for sequencing. The enriched sgRNAs were sequenced by NGS, followed by MAGeCK analysis. (B) Coomassie blue staining of indicated TcAs utilized in the BLI assay in Fig 5F. (C) The kinetic measurements of the interaction of indicated TcAs with immobilized biotin-Heparin. Kinetic parameters were obtained by fitting processed data with the 1:1 binding kinetics model (global fit). (D) Coomassie blue staining and Western blot analysis of indicated Tc toxins. All these Tc toxins are prepared by co-incubation of TcdB2-TccC3-Flag fusion protein with indicated TcAs. The autocleaved TcC C-terminal fractions (TccC3-CTR) were detected by the Flag antibody.
(TIF)

S6 Fig Schematic drawing of different TcAs. Schematic drawing of TcdA1^W14^ (green), TccA1 and TccB1 of *P*. *laumondii* (blue), and TcaA and TcaB of *Y*. *pseudotuberculosis* (orange). The N-terminal VRP1 domain (PF03538), the middle Neuraminidase domain (PF18413) and the C-terminal TcA_TcB_BD domain (PF18276) were shown with different patterns. The positions of each domain in different TcAs were shown.
(TIF)

S7 Fig The taxonomic distribution of 1,189 predicted TcC proteins. The detailed phylogenetic tree of 1,189 predicted TcA proteins as shown in Fig 6. TcA proteins encoding the RBD-D domain (PF18518) are indicated by solid red circles at the leaf. The five distinct types of TcA group are highlighted with colored shadows (key). The dbTC locus ID and related gene ID of each TcA set are provided before genome name. Middle strips are color coded by bacterial families as indicated by the key. Detailed genetic organization of each locus is provided in right schematic linear map (to scale), with the location of known protein domains highlighted as colored ellipses (key).
(PDF)

S8 Fig Sequence alignment of RBD-D of TcAs. Comparison of sequence logos of the RBD-D domain of all 322 RBD-D-containing TcAs. The logos were constructed by WebLogo with default settings.
(TIF)

S9 Fig The similarity matrix of 332 RBD-D-containing TcAs. Pairwise amino acids comparison between the RBD-D of TcAs. Each pixel in the upper triangle of the matrix color-codes sequence identity, and each pixel in the lower triangle indicate the sequence similarity.
(PDF)
