# Supplementary figures and images for: N-Glycans and sulfated glycosaminoglycans contribute to the action of diverse Tc toxins on mammalian cells
Source: PLoS Pathog. 2021 Feb 4;17(2):e1009244. doi: 10.1371/journal.ppat.1009244 (PMC7861375; doi:10.1371/journal.ppat.1009244)

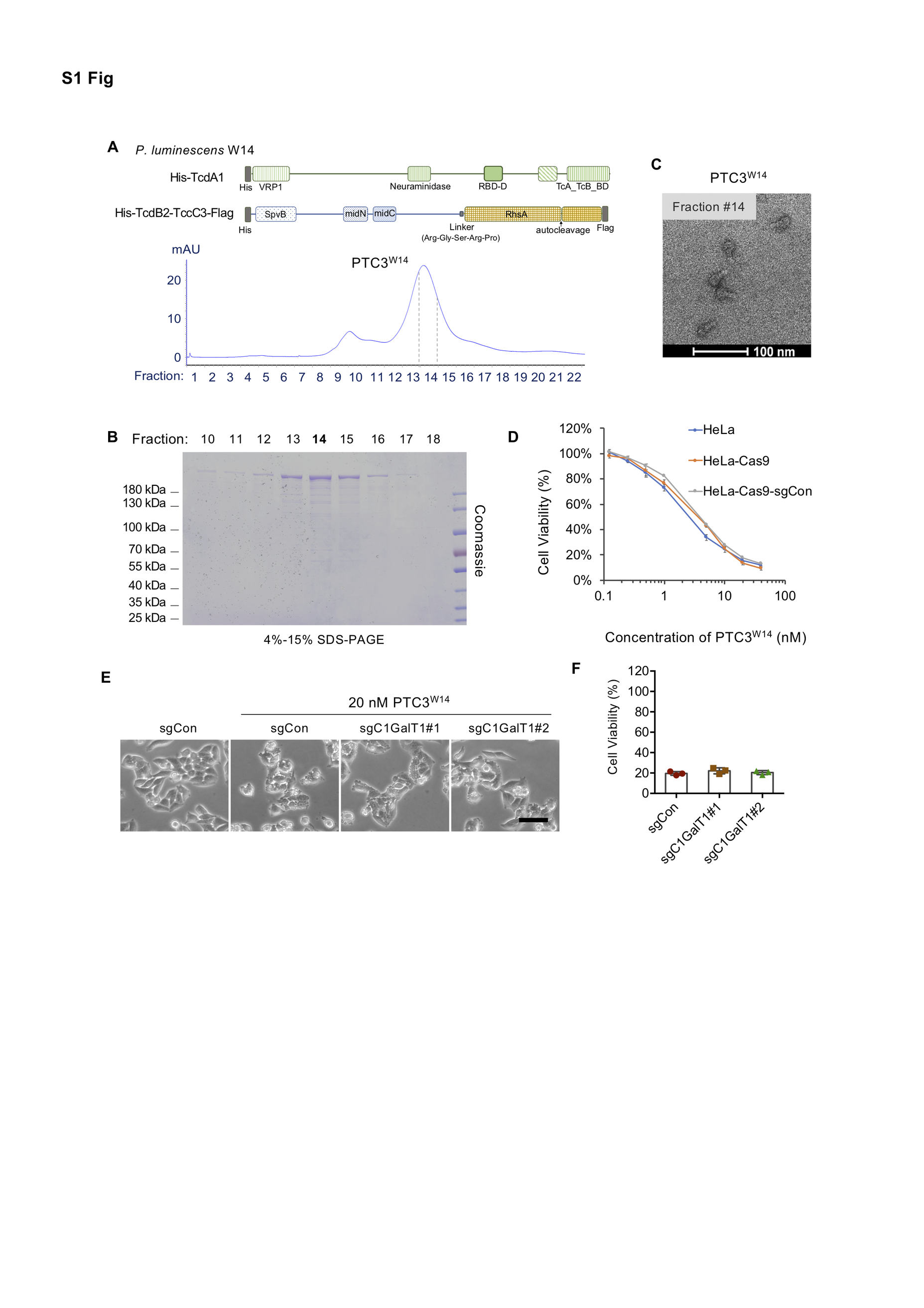

Supplement: S1 Fig — (A-B) The P. luminescens W14-derived TcdA1 and TcdB2-TccC3 fusion proteins were co-incubated at 4°C overnight, and then subjected to gel filtration analysis (A). The indicated fractions were analyzed by SDS-PAGE(B). (C) Negative stain electron micrographs of PTC3W14 (Fraction #14). Scale bars: 100 nm. (D) HeLa, HeLa-Cas9 and HeLa-Cas9-sgCon cells were treated with indicated doses of PTC3W14 for 24 h. Cell viability was measured using CCK-8 assays. (E-F) C1GalT11-KO and control HeLa-Cas9-sgCon cells (sgCon) were exposed to 20 nM PTC3W14. Representative bright field micrographs (D) and the effects on cell viability (E) were shown. (TIF) [file ppat.1009244.s004.tif]

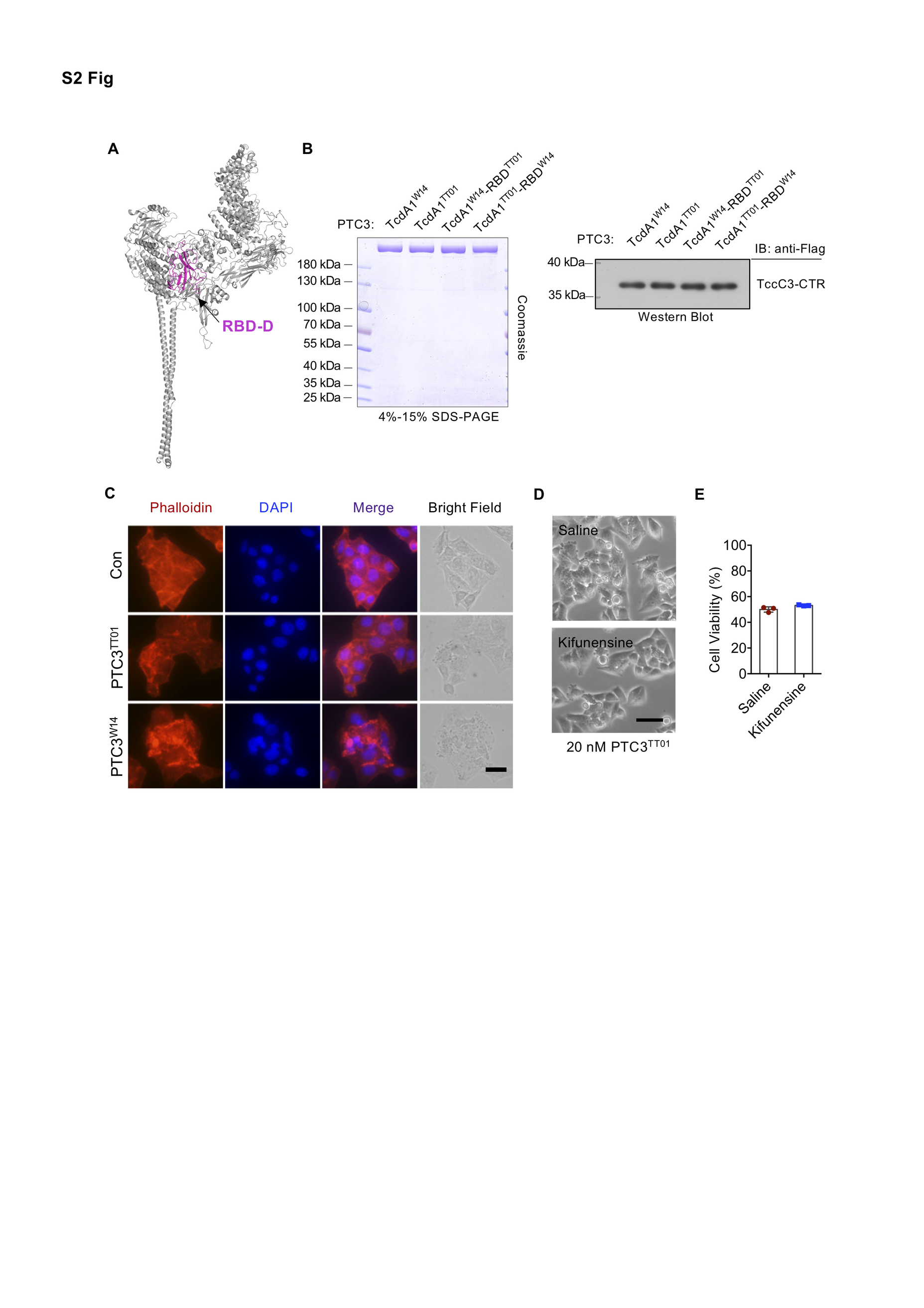

Supplement: S2 Fig — (A) Homology model of TcdA1TT01. Swiss model program was used to construct a homology model of TcdA1TT01 by alignment with TcdA1W14 (PDB: 4O9Y). The RBD-D of TcdA1TT01 were indicated in pink. (B) Coomassie blue staining and Western blot analysis of indicated Tc toxins. All these Tc toxins are prepared by co-incubation of TcdB2-TccC3-Flag fusion protein with indicated TcAs. The autocleaved TcC C-terminal fractions (TccC3-CTR) were detected by the Flag antibody. (C) HeLa-Cas9-sgCon cells were treated with 20 nM PTC3TT01 or PTC3W14 for 8 h. Cells were fixed, permeabilized and stained with with Alexa 568-phalloidin (red) and DAPI (blue). Representative fluorescence and bright field micrographs were shown. Scale bars, 5 μm. (D-E) HeLa-Cas9-sgCon cells were pretreated with saline or 20 μM Kifunensine for 12 h, then exposed to 20 nM PTC3TT01. Representative bright field micrographs were shown (D). Cell viability was measured using CCK-8 assays (E). (TIF) [file ppat.1009244.s005.tif]

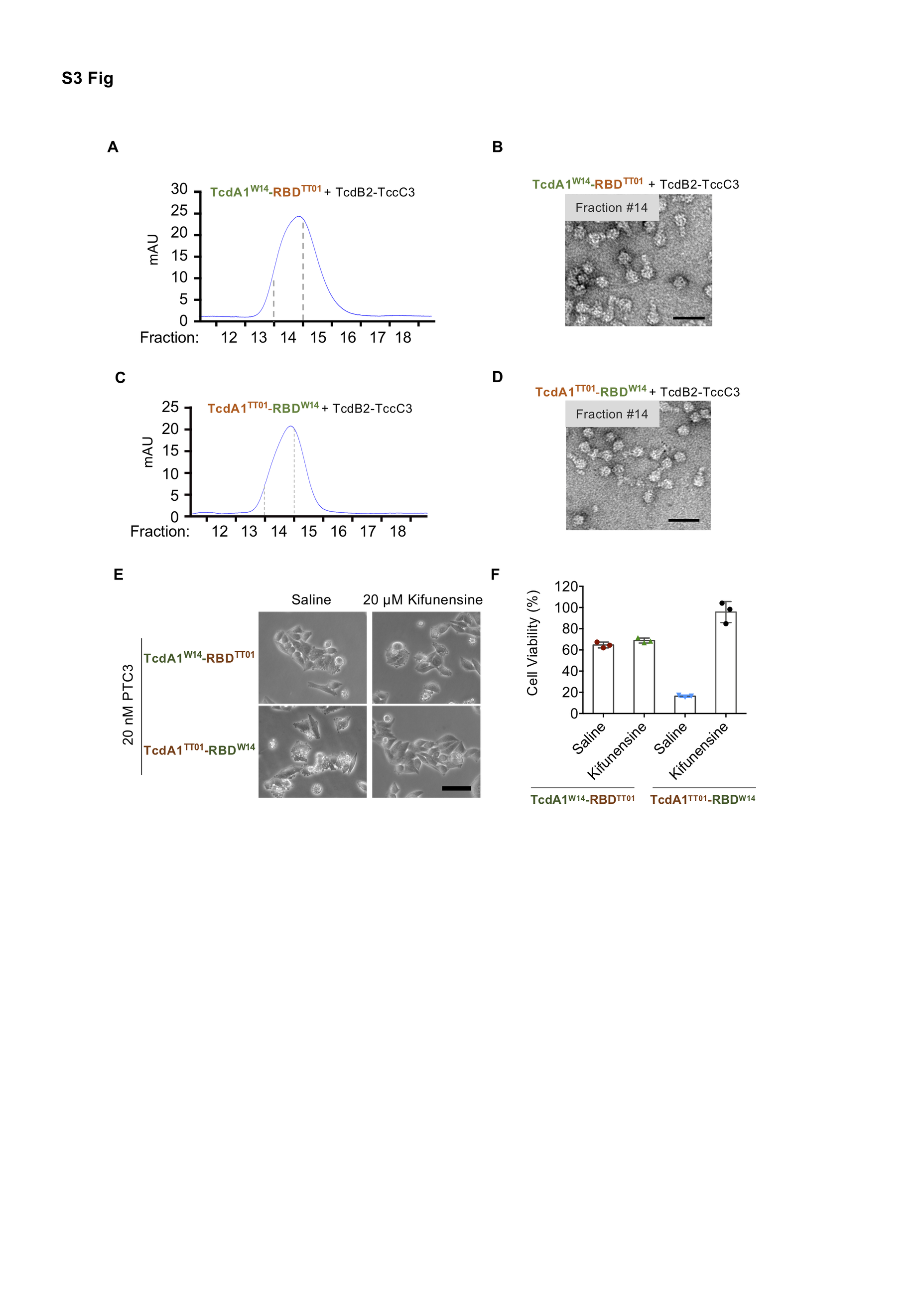

Supplement: S3 Fig — (A-D) Tc toxins formed by indicated TcAs and TcdB2-TccC3 were subjected to gel filtration analysis (A and C). Negative stain electron micrographs of the purified toxins (Fraction #14) were shown (B and D). Scale bars: 50 nm. (E-F) HeLa-Cas9-sgCon cells were pretreated with saline or 20 μM Kifunensine for 12 h, then exposed to indicated Tc toxins. Representative bright field micrographs were shown (E). Cell viability was measured using CCK-8 assays (F). (TIF) [file ppat.1009244.s006.tif]

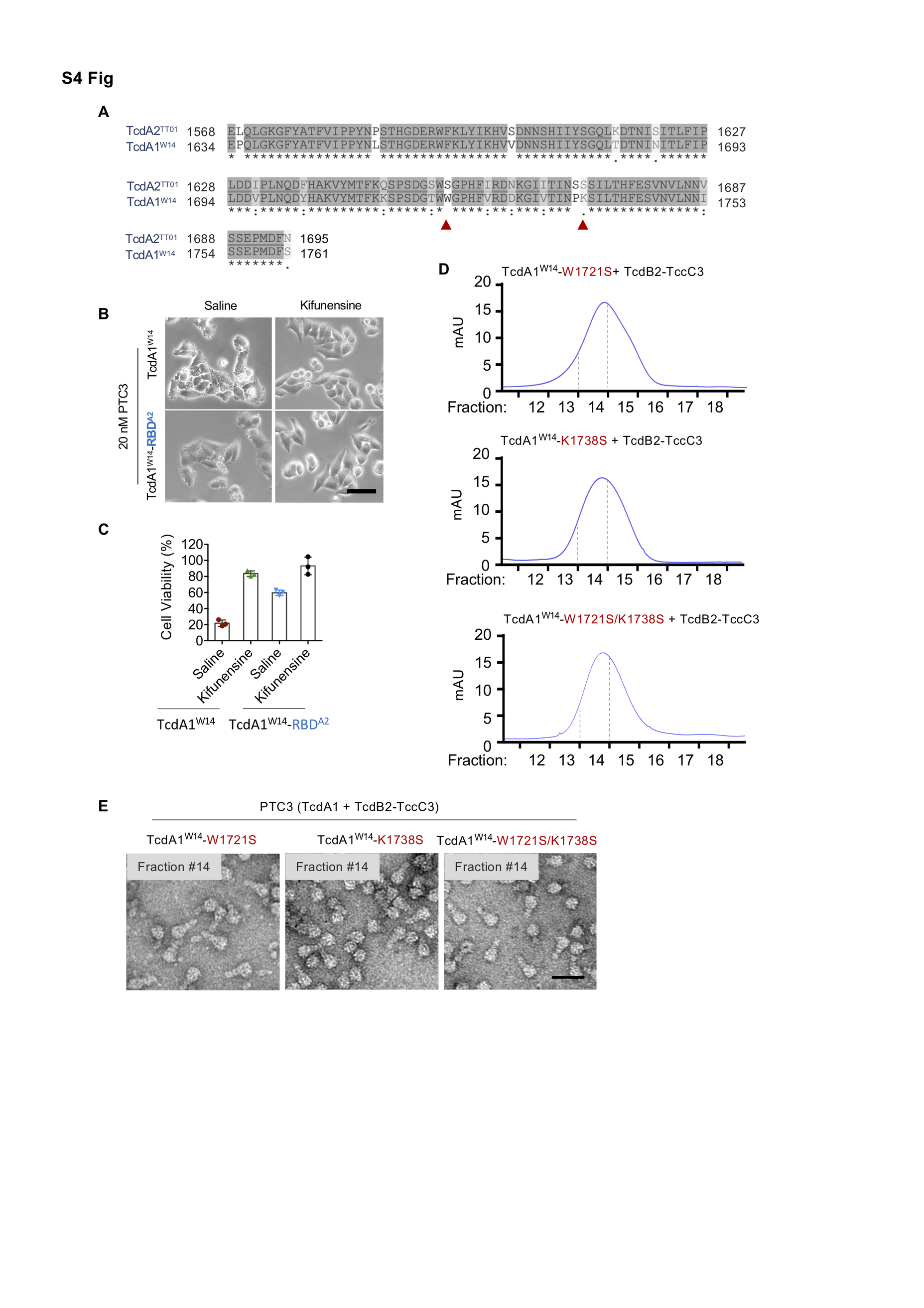

Supplement: S4 Fig — (A) Sequence alignment of the RBD-D domains derived from TcdA2TT01 or TcdA1W14, colored from minimum (white) to maximum (dark grey) conservation. The two sites that affect the binding to N-Glycans are indicated with red triangles. (B-C) HeLa-Cas9-sgCon cells were pretreated with saline or 20 μM Kifunensine for 12 h, then exposed to indicated Tc toxins. Representative bright field micrographs were shown (B). Cell viability was measured using CCK-8 assays (C). (D) Tc toxins formed by TcdA1 mutants (TcdA1W14-W1721S, TcdA1W14-K1738S and TcdA1W14-W1721S/K1738S) and TcdB2-TccC3 were subjected to gel filtration analysis. (E) Negative stain electron micrographs of the purified toxins (Fraction #14, as shown in D). Scale bars: 50 nm. (TIF) [file ppat.1009244.s007.tif]

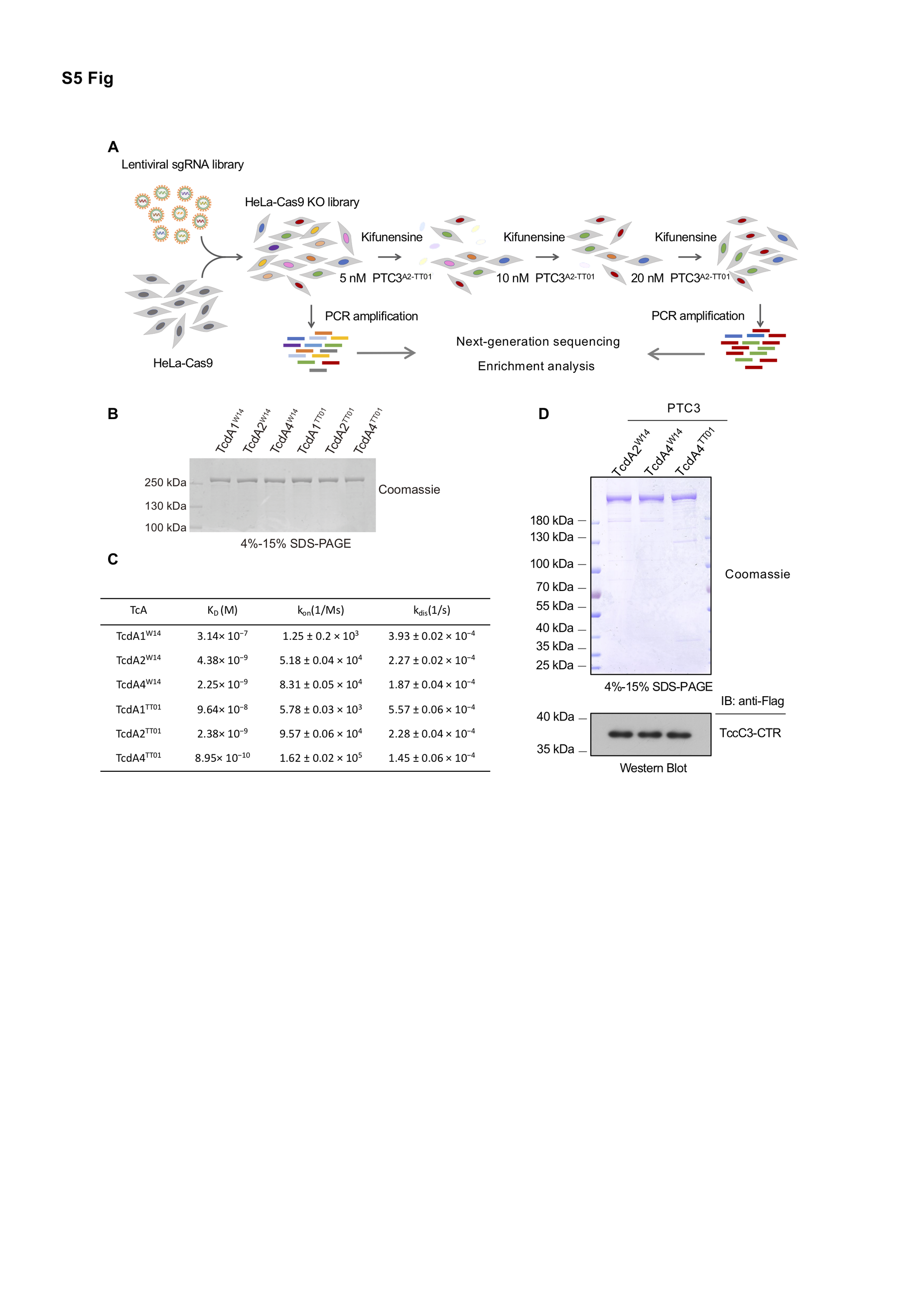

Supplement: S5 Fig — (A) Schematic drawing of the screening of PTC3A2-TT01 receptors. HeLa cells stably expressing Cas9 (HeLa-Cas9) were transduced with lentiviral GeCKO v.2 sgRNA libraries. These cells were pretreated with 20 μM Kifunensine for at least 12 h, and then exposed to increased doses of PTC3A2-TT01 (5, 10 and 20 nM). Cells that were not treated with PTC3 were served as controls. Total genomic DNA from 2×10^7^ selected and control cells was used for sequencing. The enriched sgRNAs were sequenced by NGS, followed by MAGeCK analysis. (B) Coomassie blue staining of indicated TcAs utilized in the BLI assay in Fig 5F. (C) The kinetic measurements of the interaction of indicated TcAs with immobilized biotin-Heparin. Kinetic parameters were obtained by fitting processed data with the 1:1 binding kinetics model (global fit). (D) Coomassie blue staining and Western blot analysis of indicated Tc toxins. All these Tc toxins are prepared by co-incubation of TcdB2-TccC3-Flag fusion protein with indicated TcAs. The autocleaved TcC C-terminal fractions (TccC3-CTR) were detected by the Flag antibody. (TIF) [file ppat.1009244.s008.tif]

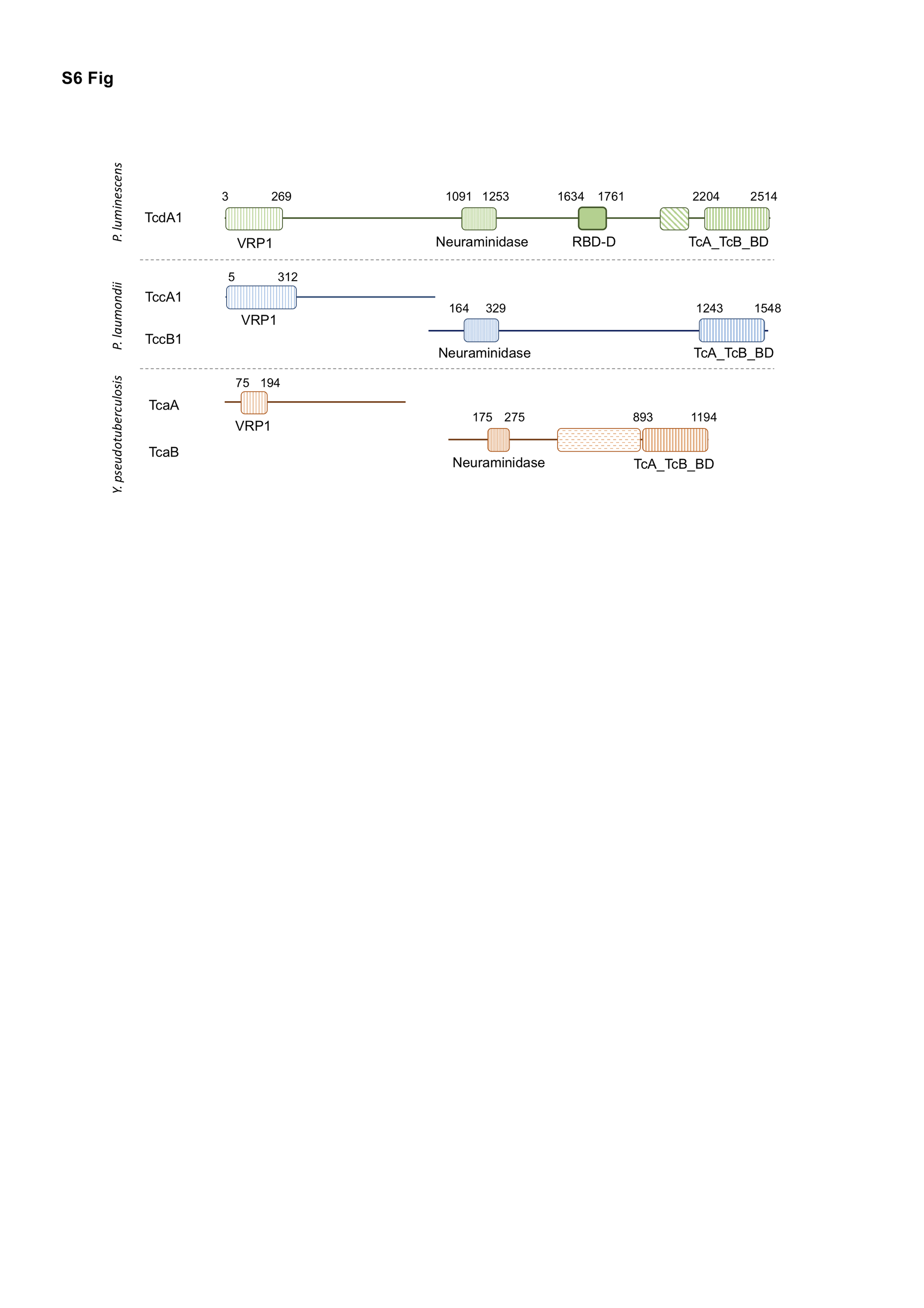

Supplement: S6 Fig — Schematic drawing of TcdA1W14 (green), TccA1 and TccB1 of P. laumondii (blue), and TcaA and TcaB of Y. pseudotuberculosis (orange). The N-terminal VRP1 domain (PF03538), the middle Neuraminidase domain (PF18413) and the C-terminal TcA_TcB_BD domain (PF18276) were shown with different patterns. The positions of each domain in different TcAs were shown. (TIF) [file ppat.1009244.s009.tif]

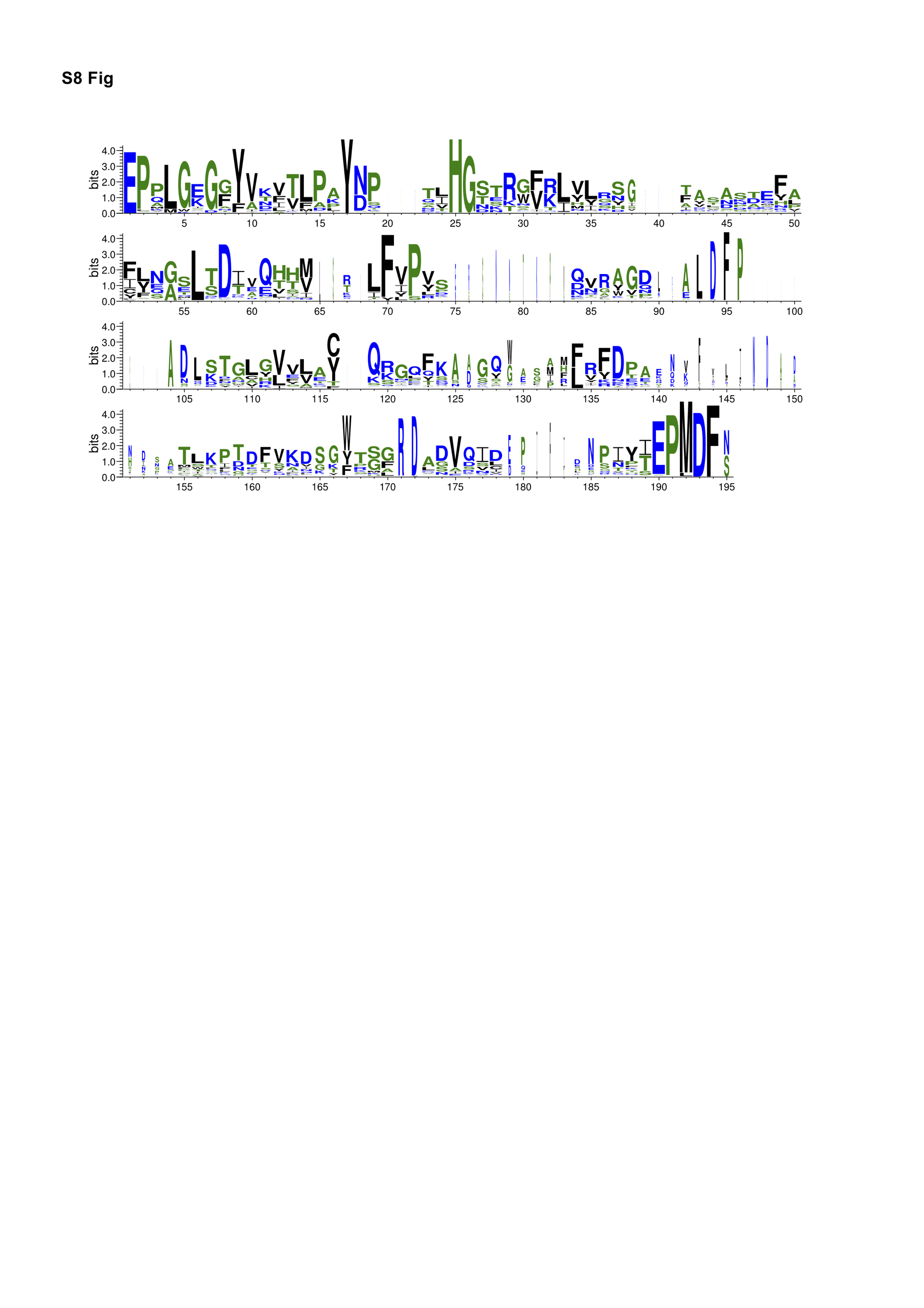

Supplement: S8 Fig — Comparison of sequence logos of the RBD-D domain of all 322 RBD-D-containing TcAs. The logos were constructed by WebLogo with default settings. (TIF) [file ppat.1009244.s011.tif]
