# Supplementary material for: N-Glycans and sulfated glycosaminoglycans contribute to the action of diverse Tc toxins on mammalian cells
Source: PLoS Pathog. 2021 Feb 4;17(2):e1009244. doi: 10.1371/journal.ppat.1009244 (PMC7861375; doi:10.1371/journal.ppat.1009244)
Supplement: S9 Fig — Pairwise amino acids comparison between the RBD-D of TcAs. Each pixel in the upper triangle of the matrix color-codes sequence identity, and each pixel in the lower triangle indicate the sequence similarity. (PDF) [file ppat.1009244.s012.pdf]

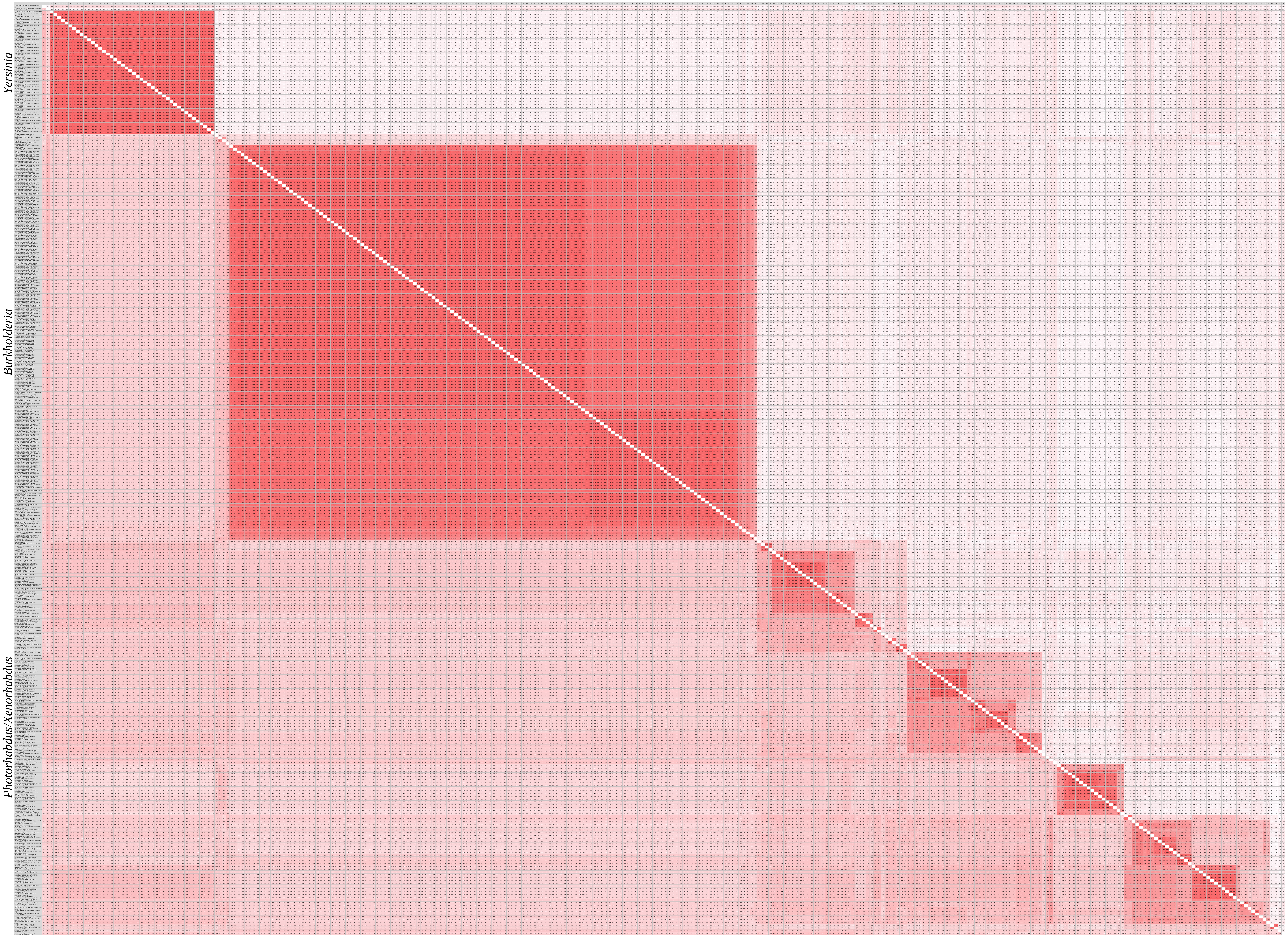

Note: pairwise amino acids comparison, upper triangle, sequence identity; lower triangle, sequence similarity.
