# Supplementary material for: N-Glycans and sulfated glycosaminoglycans contribute to the action of diverse Tc toxins on mammalian cells
Source: PLoS Pathog. 2021 Feb 4;17(2):e1009244. doi: 10.1371/journal.ppat.1009244 (PMC7861375; doi:10.1371/journal.ppat.1009244)

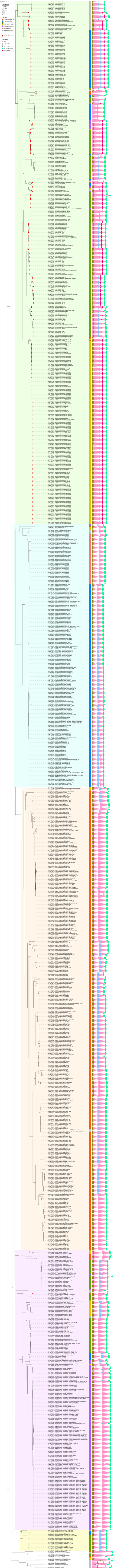

Supplement: S7 Fig — The detailed phylogenetic tree of 1,189 predicted TcA proteins as shown in Fig 6. TcA proteins encoding the RBD-D domain (PF18518) are indicated by solid red circles at the leaf. The five distinct types of TcA group are highlighted with colored shadows (key). The dbTC locus ID and related gene ID of each TcA set are provided before genome name. Middle strips are color coded by bacterial families as indicated by the key. Detailed genetic organization of each locus is provided in right schematic linear map (to scale), with the location of known protein domains highlighted as colored ellipses (key). (PDF) [file ppat.1009244.s010.pdf]
